# L-Lysine-Linked Modular Fluorescent Cholesteryl Mimics: Biophysical Properties, Molecular Interactions, and Cellular Applications

**DOI:** 10.3390/sci7020056

**Published:** 2025-05-07

**Authors:** Nicholas McInchak, Laura Stawikowska, Haylee Mesa, Jonathan Meade, Qi Zhang, Maciej J. Stawikowski

**Affiliations:** 1 Department of Chemistry and Biochemistry, Charles E. Schmidt College of Science, Florida Atlantic University, 777 Glades Rd., Boca Raton, FL 33431, USA; 2 Stiles-Nicholson Brain Institute, Florida Atlantic University, 5353 Parkside Dr., Jupiter, FL 33458, USA;

**Keywords:** cholesterol probes, fluorescence spectroscopy, membrane probes

## Abstract

Fluorescent cholesterol probes are indispensable tools for studying membrane structure, dynamics, and trafficking. To better understand the structure–function relationship of fluorescent cholesteryl probes, we developed a series of five new modular naphthalimide-containing cholesteryl probes (CND15–CND19). These probes incorporate an L-lysine linker between the cholesterol moiety and the fluorophore, along with a series of distinct head groups. We conducted extensive biophysical characterizations of these probes, including the determination of their solvatochromic properties and lipid partitioning behavior using giant unilamellar vesicles. Molecular dynamics simulations were employed to identify key molecular interactions of these probes within model lipid membranes. Furthermore, live-cell imaging in 3T3 fibroblasts demonstrated the potential applications of these analogs in live-cell imaging, measuring cellular membrane dynamics and studying cholesterol-related processes. The results of this study underscore the critical role of the linker and head group in designing fluorescent cholesterol-mimicking probes. These findings provide valuable insights into optimizing probe designs for future cholesterol and membrane biology research.

## 1. Introduction

Cholesterol is an essential lipid that plays a pivotal role in preserving cellular architecture and ensuring proper function in animal eukaryotic cells. As a key component of cell membranes, it regulates fluidity and stability, ensuring proper membrane organization and signaling [1]. In the nervous system, it is vital for synapse formation, myelin sheath integrity [2], and neurotransmission [3]. However, cholesterol imbalance has been linked to a range of cardiovascular disorders and neurodegenerative diseases, including Alzheimer’s, Huntington’s, and Parkinson’s disease [4]. Although extensive research has shed light on cholesterol’s intracellular distribution and trafficking, the exact mechanisms that govern these processes remain only partially understood [5]. By elucidating these processes, we can uncover novel targets for therapeutic intervention, ultimately leading to more effective treatments. Additionally, a deeper understanding of cholesterol trafficking could reveal fundamental insights into cellular homeostasis and the pathology of diseases associated with lipid dysregulation, thereby advancing both basic science and clinical applications.

Fluorescent cholesterol probes [6] and sensors [7,8] are indispensable tools for studying cholesterol trafficking in live cells because they enable real-time visualization, high-resolution tracking, and quantitative analysis of cholesterol dynamics. Unlike traditional biochemical methods, such as lipid extraction and mass spectrometry [9], which require cell disruption and provide only static snapshots, fluorescent probes allow continuous monitoring of cholesterol movement in living systems.

Genetically encoded cholesterol sensors and chemical fluorescent probes each have unique advantages and limitations when studying cholesterol trafficking in live cells. Due to the unique nature of cholesterol, there is no universal fluorescent cholesterol probe. The choice between genetically encoded cholesterol reporters and chemical fluorescent probes depends on the research question [10].

**Figure 1.**
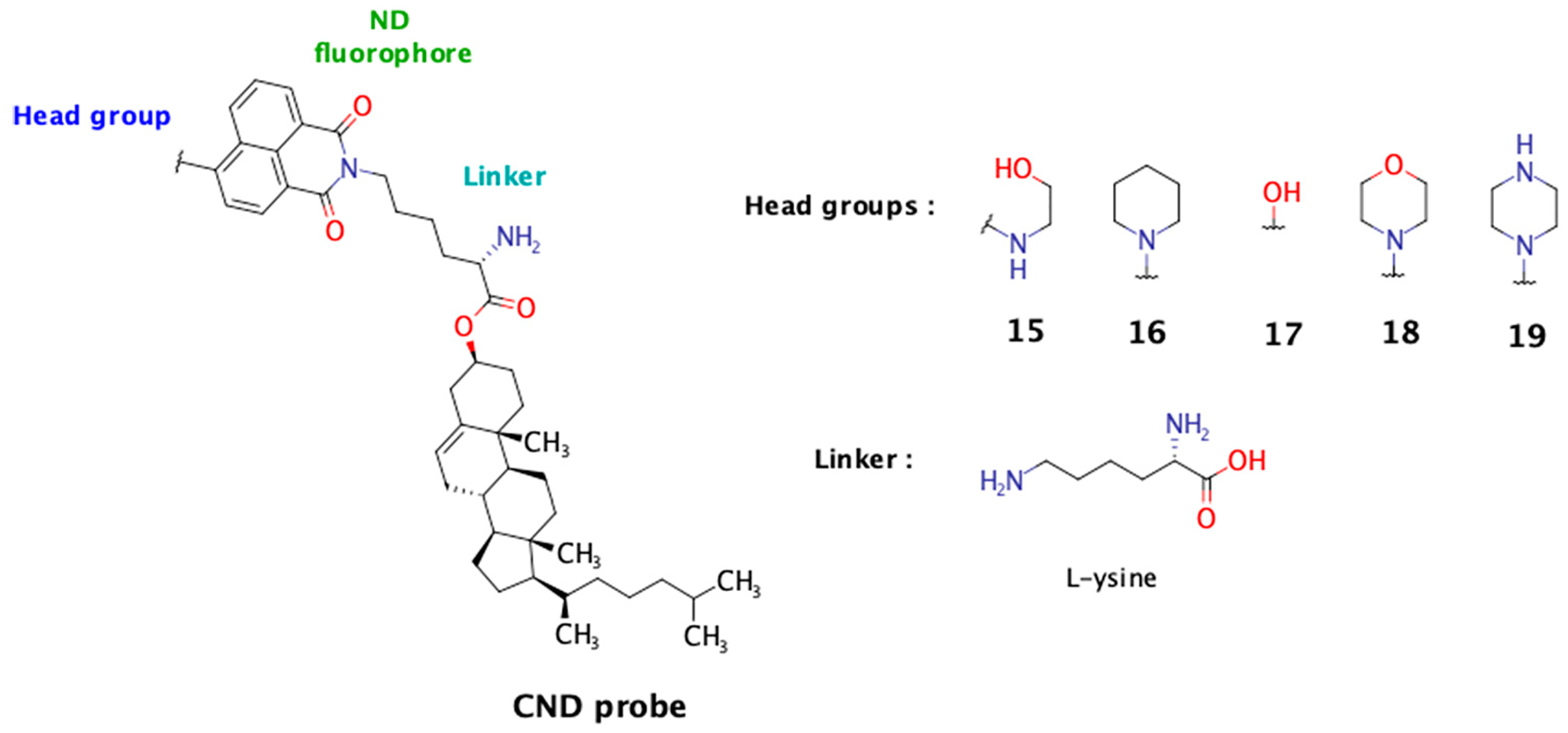
Chemical structures of CND probes with different head groups (CND15–CND19). The core structure consists of an ND fluorophore conjugated to a cholesterol analog via an L-lysine linker. The head groups vary among the probes, influencing their biophysical properties and interactions with lipid environments. Structures of the head groups for CND15 to CND19 are shown on the right.

Among existing fluorescent cholesterol probes, the fluorescent cholesteryl probes based on 1,8-naphthalimide scaffold (CNDs) recently developed in our group demonstrate excellent solvatochromic properties, partitioning into Lo phases in model membranes, and some of them are also pH sensitive [11]. The modular structure of these probes allows us to investigate the influence of different linkers and head groups on their characteristics. In a previous study, among the tested linkers (Gly, L-Ser, and β-Ala), the polar linker L-Ser enhanced probe solubility and membrane immersion and favored a desirable cholesterol tilt angle. In this study, we present our findings on five new CND analogs incorporating the L-Lys linker. This linker was selected for its unique structural features: its carboxyl group anchors it to cholesterol, the ε-amino group provides an attachment point for the naphthalimide fluorophore, and the alpha-amino group remains free and ionizable under physiological conditions. In this study, we aimed to evaluate the influence of the free alpha-amino group on the probe’s biophysical properties and compare its performance to that of the previously studied hydroxyl group from the L-serine linker. The fluorescence of the 1,8-naphthalimide probe is influenced by the nature of the substituent at the C-4 position, known as the “head group”. This moiety also plays a crucial role in determining the probe’s biophysical properties (Figure 1).

## 2. Materials and Methods

All reagents and solvents (including anhydrous ones) were obtained from commercial sources (Fisher Scientific, Waltham, MA, USA) and used without purification unless stated otherwise. The LysoView^™^ 650 was purchased from Biotium (Fremont, CA, USA), and TopFluor-Cholesterol (TFC) was purchased from Avanti Polar Lipids (Alabaster, AL, USA).

### 2.1. Thin-Layer and Column Chromatography

Thin-layer chromatography (TLC) was performed on aluminum-backed plates coated with silica gel (60 Å pore diameter, 200 μm layer thickness; SiliCycle (Quebec, QC, Canada) or EMD Millipore (Merck Millipore, Burlington, VT, USA). All TLC plates contained a fluorescent indicator and were visualized under UV light (λ_ex = 254 nm or 366 nm) or by staining with a solution of 10% H_2_SO_4_ in ethanol followed by brief heating with a heat gun. Column chromatography was conducted using columns packed with silica (60 Å pore diameter, 40–60 μm particle size; SiliCycle).

### 2.2. NMR and Mass Spectrometry

NMR spectra were recorded on 400 MHz spectrometer at 22 °C in *d*-chloroform or *d*_4_-methanol. Proton (^1^H) and carbon (^13^C) signals were referenced to tetramethylsilane (TMS), and signal multiplicities are denoted as singlet (s), doublet (d), triplet (t), quartet (q), pentet (p), multiplet (m), broad (br), or combinations thereof. Mass spectra were obtained using a Bruker Microflex MALDI-TOF spectrometer (Bruker Corporation, Billerca, MA, USA) with α-cyano-4-hydroxycinnamic acid (α-CHCA) as the matrix and were recorded in positive ion mode.

### 2.3. Synthesis of CND Probes

The synthesis scheme and detailed methods for preparing the CND probes are provided in the Supplementary Material.

### 2.4. Determination of Absorbance Spectra in Organic Solvents

Stock solutions of the CND probes were diluted to 10 μM in chloroform and evaporated to dryness. The residues were re-dissolved in dichloromethane, dimethyl sulfoxide, ethanol, or hexane to yield 10 μM solutions. Absorbance spectra were recorded from 300 to 700 nm using a Thermo Scientific Evolution 201 UV–VIS spectrophotometer (Thermo Scientific, Waltham, MA, USA).

### 2.5. Determination of Emission Spectra in Organic Solvents

Stock solutions of the CND probes were diluted to 1 μM in chloroform and evaporated to dryness. The residues were re-dissolved in the respective solvents to yield 1 μM solutions. Emission spectra were recorded using a fixed excitation wavelength of 400 nm on a PerkinElmer LS 55 fluorescence spectrometer (PerkinElmer, Waltham, MA, USA), scanning from 420 to 650 nm.

### 2.6. Determination of Molar Extinction Coefficients in DMSO and CHCl_3_

Stock solutions of the CND probes were prepared at 100 μM in the respective solvents. For each data point, the solutions were serially diluted by a factor of 0.75 in triplicate. Absorbance values were recorded at the wavelength corresponding to the absorbance maximum for each solvent using a Thermo Scientific Evolution 201 UV–VIS spectrophotometer.

### 2.7. Fluorescence Dependence on pH in 1% Octyl-β-Glucoside (OG) Solution

OG solutions (1%) were prepared at pH values ranging from 5 to 10 using Sorenson’s phosphate buffer. After gentle mixing, the solutions were allowed to equilibrate for 30 min. A stock solution of the CND probes in DMSO was diluted to 20 μM. For each pH, 95 μL of the OG solution was added to a well in a 100 μL well plate, followed by 5 μL of dye solution, with the solution aspirated three times. The plate was then incubated in the darkness for 15 min. Fluorescence measurements were conducted using a Spectramax Gemini EM plate reader (Molecular Devices, San Jose, CA, USA) with a fixed excitation wavelength of 405 nm, scanning the visible range with an excitation cut-off at 455 nm. Fluorescence values at the emission maximum were plotted against pH.

### 2.8. Preparation and Analysis of Lipid Phase Partitioning in Giant Unilamellar Vesicles (GUVs)

Lipid stock solutions of DOPC (10 mM), brain sphingomyelin (10 mM), cholesterol (10 mM), DiD (100 μM), and CND dye (40 μM) were prepared in a 9:1 CHCl_3_:MeOH mixture, and the GUVs were formed in water containing 50 mM sucrose as described previously [11] using the Vesicle Prep Pro (VPP) instrument (Nanion Technologies, Munich, Germany). GUV images were acquired on a Nikon A1R confocal microscope (Nikon, Melville, USA) with a 20× objective. CND detection was performed with a 405 nm excitation and an emission range of 500–550 nm, while DiD was excited at 640 nm with an emission range of 663–738 nm. Individual vesicle images were cropped from the raw data, and vesicles displaying clear phase separation in the DiD channel were analyzed using the GUV-AP plugin to quantify liquid-ordered (Lo) partitioning [12]. Vesicle identification and stitching were performed using the DiD channel with a threshold coefficient of 0.12, a circle extension coefficient of 1.2, and an angle step of 3°. The fraction of probes present in the Lo phase was calculated according to the following equation:

$$\%\text{Lo}=\frac{\text{FLo}}{\text{FLo}+\text{FLd}}\times 100$$

where FLo represents the average fluorescence intensity of the circular segment corresponding to the Lo phase, and FLd represents the average fluorescence intensity of the circular segment corresponding to the Ld phase.

### 2.9. Molecular Dynamics Simulations and Analysis

CND probe molecules were constructed using the CHARM-GUI server’s input generator, ligand reader, and modeler modules [13–15]. A positive charge was added to the piperazine group as well as α-amino group of lysine to represent its protonation state at physiological pH. CHARMM-compatible topology and parameter files were generated using CGenFF parametrization protocol [16]. Membrane structures were built using the membrane builder, positioning the probe such that the naphthalimide moiety resided at the membrane surface and cholesterol was embedded within the hydrophobic core [17]. The membrane model consisted of 25% sphingomyelin (SSM), 25% cholesterol, and 50% POPC, with lipids symmetrically distributed between leaflets. Neutral pH and physiological salt conditions (0.15 M NaCl) were maintained, and the systems were charge neutral. Molecular dynamics simulations were carried out using the CHARMM36m force field and the GROMACS 2022.1 package with GPU acceleration [18]. Following system construction, energy minimization was performed using the steepest descent method with hydrogen bond constraints using the LINCS algorithm [19]. The system underwent six equilibration phases at 298.15 K as per the CHARMM-GUI protocol, followed by a 200 ns production run in the NPT ensemble using the Nose–Hoover thermostat. Electrostatics were calculated using the particle mesh Ewald method with a 1.2 nm cut-off. The final 150 ns of each trajectory were used for analysis. Trajectory data were processed in Visual Molecular Dynamics (VMD, ver. 1.9.4) [20], and membrane properties such as cholesterol tilt and thickness were evaluated using the MembPlugin [21]. Bilayer thickness was determined from the positions of phosphorus atoms, while cholesterol tilt angles were calculated by analyzing the positions of the C10 and C13 carbons relative to the membrane normal defined by the phosphorus atom mass distribution. Immersion values were determined by calculating the distance of selected oxygen atoms (the C-3 bound oxygen and, where applicable, the hydroxyl oxygen) from the membrane center, defined as the average of the phosphorus atom positions in each leaflet. Hydrogen bonds between the probe and membrane components (POPC, sphingomyelin, water) were quantified using VMD’s H-bond plugin with a donor-acceptor distance cut-off of 3 Å and an angle cut-off of 30°. These analyses were performed on every frame of the final 150 ns of the trajectories. A total hydrogen bond count is reported for all CNDs. For cholesterol, the hydrogen bonds are presented as an average that is calculated by dividing the total number of bonds by the number of cholesterol molecules, providing a per molecule value. Representative CND structures were obtained with clustering analysis using UCSF Chimera (ver. 1.16) [22], with coordinates extracted from the top ensemble representative frame.

### 2.10. Cell Culture

NIH-3T3 fibroblasts were obtained from ATCC (ATCC, Manassas, VA, USA). A vial of cryopreserved cells was thawed in a 37 °C water bath and immediately plated in 75 mL culture flasks pre-coated with Matrigel (Corning, Corning, NY, USA). Cells were cultured to confluence in DMEM supplemented with GlutaMax and 5% fetal bovine serum. Confluent cultures were washed with PBS and detached using 1% Trypsin/EDTA. Following centrifugation, the cell pellets were resuspended in the same media and plated onto quad-divided glass-bottom 35 mm culture dishes at a density of 1,000,000 cells/cm^2^. Cultures were maintained in the same media and used within 2–5 days.

### 2.11. Live-Cell Fluorescence Confocal Imaging

Live-cell imaging was performed using a Nikon A1R confocal microscope equipped with a 60× (N.A. 1.40) oil immersion objective and excitation lasers with appropriate filters for CNDs (405 nm excitation), TopFluor-Cholesterol (TFC) (488 nm excitation), and LysoView 650 (640 nm excitation). Image acquisition was carried out using Nikon Elements, with laser power, gain, and offset settings optimized to maximize dynamic range while minimizing fluorophore bleed-through; these settings were maintained consistently across experiments. Cells were incubated with the desired probe to a final concentration of 1 μM for 1 h, followed by incubation in dye-free media for the required time period. Prior to imaging, cells were loaded with 0.5 μM LysoView 650 for 1 h, then washed with dye-free media, and placed in an Oko-Lab weather chamber (Okolab, Pozzuoli, Italy) mounted on the microscope stage. The chamber and the 60× objective were pre-warmed to 37 °C, and the chamber was maintained at 5% CO_2_ and 100% humidity. For each sample, three random fields of view were captured at a resolution of 1024 × 1024 pixels. Z-stack images were acquired with a step size of 0.3 μm, and time-lapse imaging was performed with a 10-s frame interval over 3–5 min. Image analysis was subsequently performed using ImageJ/Fiji (ver. 2.16.0) [23].

### 2.12. Particle Analysis

Fluorescence intensity across Z-stacks was evaluated, and a representative frame (with high fluorescence intensity) was extracted for further analysis after applying the “despeckle” (median) filter. Regions of interest (ROIs) were generated using trainable Weka segmentation [24]. The classifier was trained on series of low signal-to-noise and high signal-to-noise images. Classified images were converted to masks, and ROIs were subsequently outlined. Particle analysis was conducted with Fiji/ImageJ, using unprocessed images with object detection limited to areas between 0.1 μm^2^ and 10 μm^2^. For each ROI, the mean pixel intensity and area were determined, and particle diameters were estimated assuming circular geometry. Further analysis was limited to particles ranging from 0.3 to 1 μm. Data from 1-h and 24-h time points were compared and plotted using GraphPad Prism (version 10), with statistical significance assessed using the Wilcoxon matched pairs signed rank test.

### 2.13. Colocalization Analysis

Pearson’s correlation coefficient (PCC) and Manders’ colocalization coefficient (MCC) values [25] were determined using the EzColocalization plugin in Fiji/ImageJ [26]. Colocalization was performed on single frames from the Z-stack (selected based on maximum green channel intensity) with Costes’ thresholding. PCC and MCC values were obtained for each analog in triplicate. Data from 1-h and 24-h experiments were compared and plotted using GraphPad Prism (version 10), and statistical analysis was conducted using the Wilcoxon matched pairs signed rank test.

### 2.14. Visualization and Drawing Software

Reaction schemes and 2D structural formulas were prepared using MarvinSketch (ChemAxon Ltd., Budapest, Hungary, version 21.15, https://www.chemaxon.com, accessed on 1 March 2025), while three-dimensional molecular visualizations were generated with UCSF Chimera (version 1.16) [22]. Some plots were obtained using RStudio (version 2024.04).

## 3. Results

### 3.1. Synthesis of CND Probes

The fluorophore core of CND probes consists of a bifunctional 1,8-naphthalimide scaffold, a push-pull fluorophore whose optical properties are strongly influenced by the electron-rich C-4 substituent (head group) [27]. This design imparts excellent solvatochromic properties, making it highly sensitive to environmental changes [28]. In this study, we synthesized five new CND analogs (CND15–CND19) featuring distinct head groups and an L-lysine linker, which ties the fluorophore to the cholesterol moiety (Figure 1). The probes were obtained through a four-step synthetic route, following an established strategy (Scheme S1) [11]. The choice of the L-lysine linker was driven by previous findings that the hydroxyl group of the L-serine linker affected probe membrane immersion by interacting with water and neighboring lipids. Based on this, we hypothesized that the L-lysine linker might exhibit similar behaviors. Additionally, the positively charged α-amino group under physiological conditions could further influence lipid ordering and enhance cellular uptake.

### 3.2. Solvatochromic Characterization

The incorporation of the L-Lys linker did not significantly alter the molar extinction coefficients measured in chloroform and DMSO; these values remained consistent with those of other CNDs featuring the same head group type published previously [11] (Table 1).

The absorption properties of the five CND analogs were assessed in chloroform and DMSO, revealing notable variations in molar extinction coefficient (ε) and absorbance profiles (λ_max_). These results indicate that solvent polarity significantly influences both the absorption intensity and spectral position, with red shifts observed in DMSO due to its higher polarity compared to chloroform. CND15 exhibited high ε and the longest λ_max_ in both solvents, suggesting stronger electronic transitions, while CND17 showed the lowest ε and the most blue-shifted λ_max_, indicative of weaker conjugation.

The solvatochromic properties of CND15–CND19 were further investigated using hexane, dichloromethane, ethanol, and DMSO as solvents. The data demonstrate classic signs of solvatochromism in the 1,8-naphthalimide-based dyes (Figure S1). For all dyes, the main absorption band moves to longer wavelengths (red-shifts) as the solvent polarity increases from hexane (least polar) to DMSO (most polar). This behavior is typical of dyes with an intramolecular charge transfer (ICT) character in their excited state [29]. The more polar the solvent is, the stronger the stabilization of that ICT state, causing a red shift in the absorption maximum [30]. The maximum absorbance (λ_max_) varies in different solvents. This may reflect changes in the molar absorptivity of the dye in different solvents or other solvent-specific effects [31].

The fluorescence spectra (Figure S1) show that the emission peaks shift depending on the solvent polarity, with polar solvents often causing a red shift due to better stabilization of the excited state. Additionally, fluorescence intensity varies with solvent polarity, where specific interactions can either enhance or quench the emission. These behaviors indicate that the CND dyes possess a significant intramolecular charge transfer character, making their excited states highly sensitive to changes in the solvent environment.

**Figure 2.**
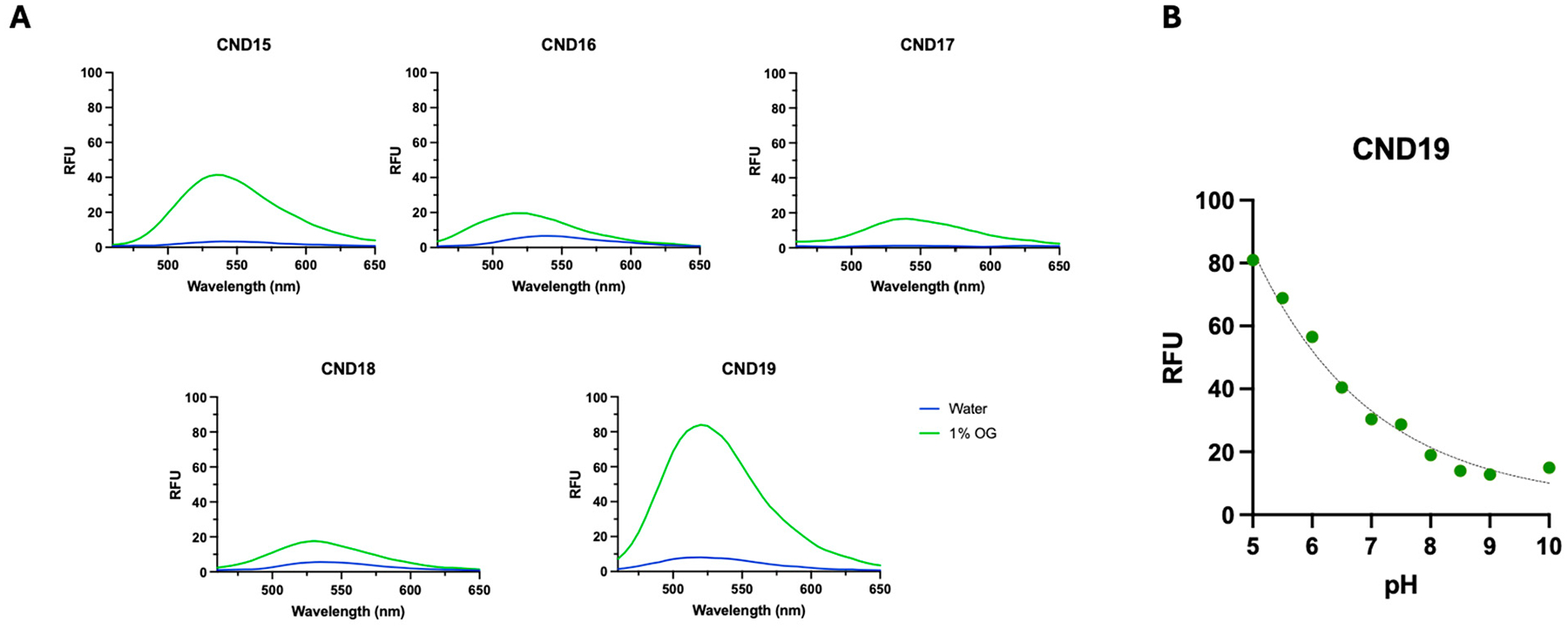
Fluorescence properties of CND probes in different environments and pH dependence of CND19. (**A**) Emission spectra of CND15–CND19 in water (blue) and 1% octyl glucoside (OG) micelles (green). The fluorescence intensity increases significantly in the micellar environment. (**B**) pH-dependent fluorescence response of CND19, showing a ~2.5-fold increase in relative fluorescence as pH decreases from 7 to 5, confirming that protonation of the piperazine head group affects its fluorescence properties.

By incorporating a linker with a free α-amino group into the probe structure, the probes exhibited increased hydrophilicity, consequently reducing their solubility in non-polar organic solvents. To evaluate the solvatochromic properties and membrane-sensing capabilities of CND15–CND19, we compared their fluorescence spectra in octyl glucoside (OG) micellar solutions, a surrogate for membrane environments, with those in water. Spectra were recorded by adding a DMSO stock solution of each probe to either OG micellar solutions or water. Probes with polar head groups (CND15, CND17, and CND19) exhibited the most significant differences in fluorescence intensity between OG and water (Figure 2A). This result suggests that these dyes have a lower tendency to precipitate in water compared to their neutral analogs, CND16 and CND18. Additionally, CND16–18 exhibit a slight hypsochromic shift when comparing spectra in water and OG, whereas CND15 and CND19 show a minor bathochromic shift, consistent with the solvatochromic behavior observed in organic solvents. The CND19 analog, featuring a piperazine moiety at the head group position, also exhibited pH-dependent fluorescence (Figure 2B), consistent with other CND analogs possessing the same head group. The fluorescence intensity increases approximately 2.5-fold as the pH decreases from 7 to 5.

### 3.3. Lipid Partitioning

**Figure 3.**
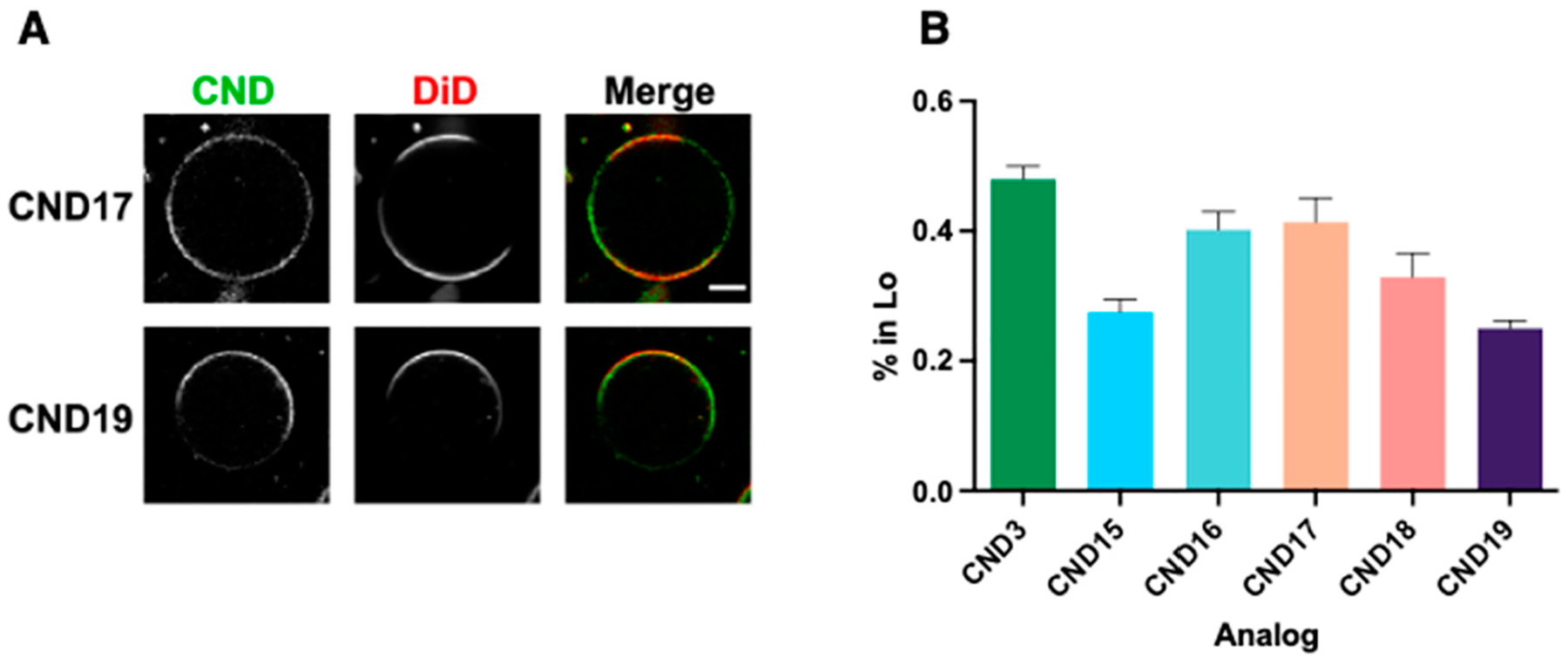
Partitioning of CNDs between Ld/Lo phases in phase-separated GUVs. (**A**) Representative fluorescent confocal image of CND17 and CND19 together with the Ld marker DiD. Scale bar—10 μm. (**B**) Fraction of each CND analog partitioning into the Lo phase, determined by measuring fluorescence intensity along the vesicle perimeter using the GUV-AP plugin for ImageJ. Data are represented as mean ± SEM.

The Lo/Ld partitioning properties were evaluated using phase-separated giant unilamellar vesicles (GUVs), prepared from a DOPC:SM:Chol (2:2:1) lipid mixture using electroformation with a previously established protocol [11]. CND dyes and the Ld phase marker (DiD) were incorporated at a 0.1 mol% concentration. The results, presented in Figure 3, were compared to the previously reported best-performing CND3 analog. Among the tested analogs, CND17 and CND16 exhibited the highest Lo phase partitioning (~40%), while the most polar analog, CND19, showed the lowest Lo phase preference (~25%), highlighting the influence of polarity on membrane domain localization. These results qualitatively align with previous findings on the influence of head groups in different linker variants on Lo phase partitioning.

### 3.4. Molecular Dynamics Simulations

**Figure 4.**
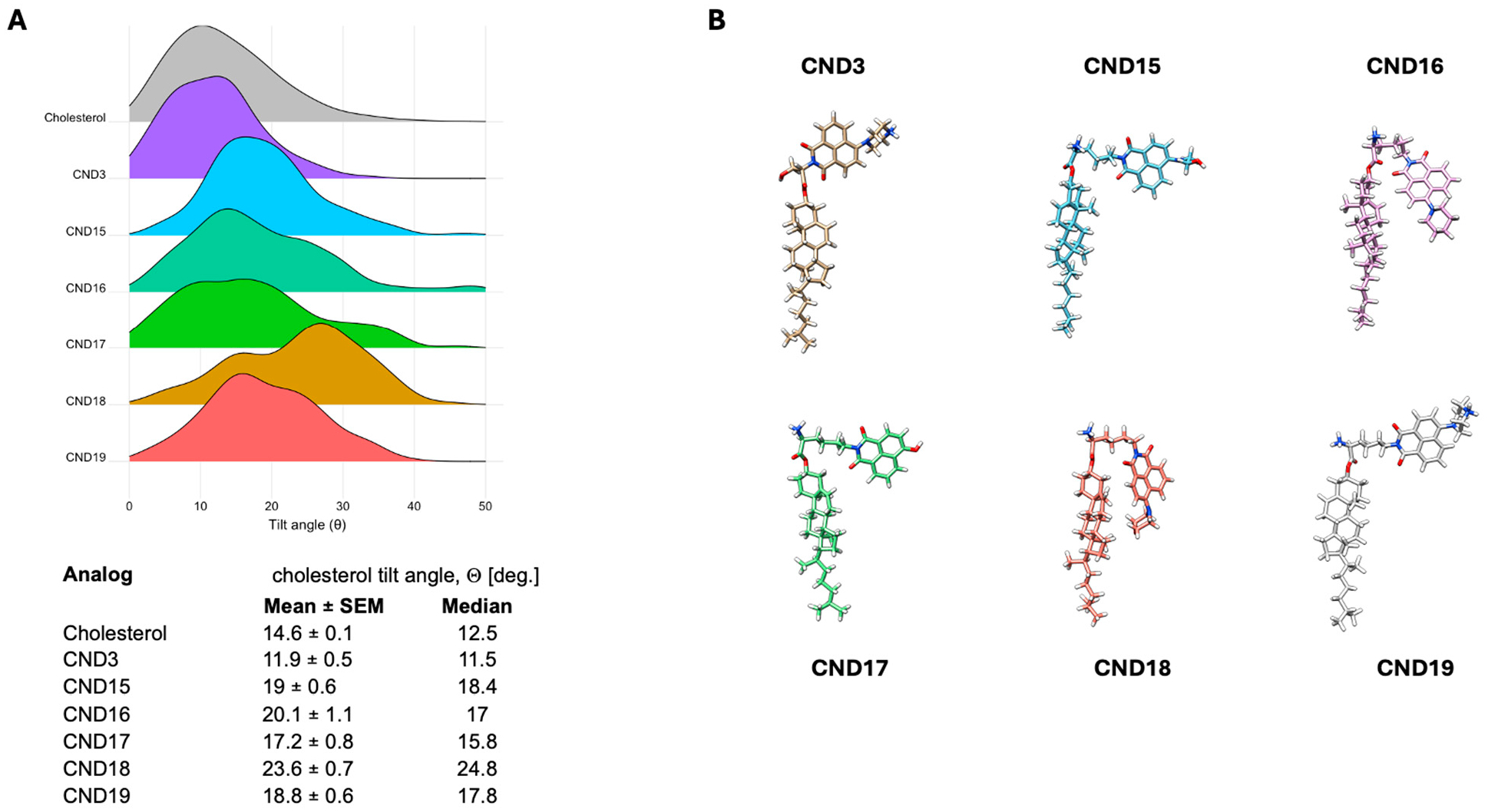
Cholesterol tilt angle distributions and structural comparison of CND analogs. (**A**) Probability distributions of the sterol tilt angle for cholesterol (gray) and CND3–CND19 (colored). The mean ± SEM and median tilt angles are summarized in the table. (**B**) Representative molecular structures of CND15–CND19 obtained from MD trajectory cluster analysis compared with CND3, illustrating the ND fluorophore and linker positions relative to cholesterol.

Our objective is to gain a more comprehensive understanding of the complex molecular interactions that CND probes may experience within a lipid environment. To accomplish this, we performed molecular dynamics simulations using a POPC:SM:Cholesterol system at a 2:1:1 ratio, representing the cholesterol concentration typically found in plasma membranes. These simulations not only provide insights into the behavior of CND probes under biologically relevant conditions but also serve as a valuable tool for guiding the design of improved probes with enhanced properties. CND15–CND19 systems were constructed with the probe embedded in the membrane, positioning the O3 atom of cholesterol at the level of the phosphate groups. The production simulation was run for 200 ns, with the CND3 probe and cholesterol serving as reference points for comparison. The probes were evaluated based on key parameters, including cholesterol tilt angle, membrane immersion depth, and their propensity to form hydrogen bonds with neighboring lipids and water molecules. The cholesterol tilt angle (θ) was determined using a vector defined by the positions of the C10 and C13 atoms in cholesterol, measuring the angle it forms with the membrane normal (Z-axis). The overall cholesterol tilt angle is strongly influenced by interactions between the conjugated fluorophore and its local microenvironment (Figure 4). The data reveal that the tilt angle increases in the following order: CND17 < CND19 < CND15 < CND16 < CND18. Here, the latter analogs exhibit substantially higher angles than both native cholesterol and CND3. Notably, the hydrophobic head groups in CND16 and CND18 promote a downward orientation toward the membrane core, which likely accounts for their increased tilt angles. These findings confirm that modifications to the fluorophore can significantly alter cholesterol orientation within the membrane, potentially impacting membrane properties and interactions.

**Figure 5.**
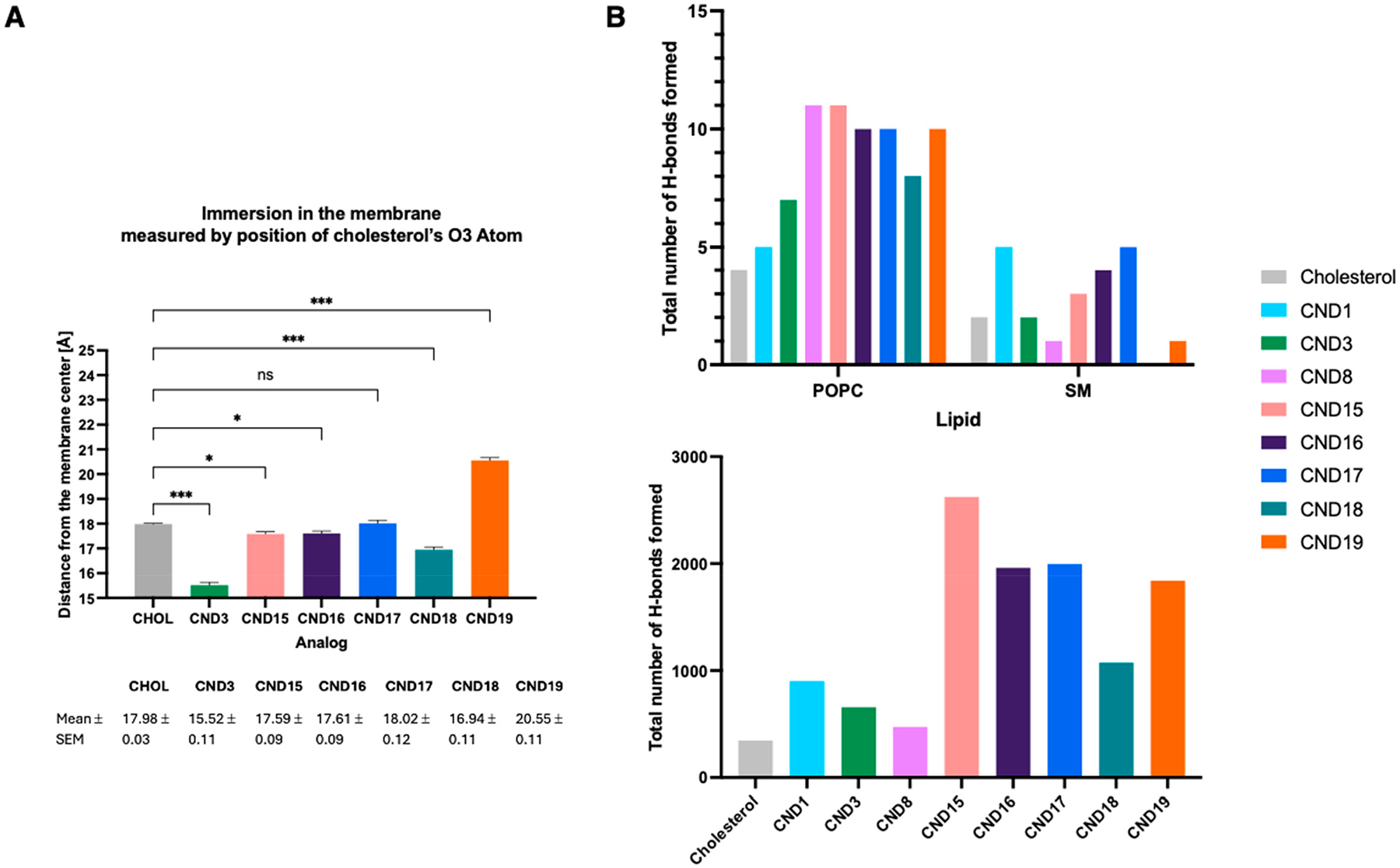
Characterization of CND probe behavior using MD simulations, with direct comparison to CND3. (**A**) Immersion depth of cholesterol (CHOL) and CND analogs in the membrane, measured by the position of the sterol O3 atom relative to the bilayer center. Data are represented as mean ± SEM. Statistical significance was assessed using a one-way ANOVA followed by Dunnett’s multiple comparison test to compare each CND analog to the CHOL control. Significance is indicated as follows: *p* < 0.05 (*), *p* < 0.01 (**), *p* < 0.001 (***); ns, not significant. (**B**) Analysis of hydrogen bonds formed by cholesterol and CND analogs with neighboring lipids (top panel) and water (bottom panel), illustrating how lysine linker and head group modifications affect membrane interactions.

Cholesterol’s hydroxyl group plays a pivotal role in lipid ordering, hydrogen bonding, and lipid interactions, making it a key determinant of membrane structure and dynamics [32]. To evaluate how the CND probes compare with cholesterol in this regard, we measured their immersion depth by tracking the position of cholesterol’s O3 oxygen atom throughout the trajectory. Figure 5A shows that CND15–17 embed themselves in the bilayer at roughly the same depth as cholesterol. Within this series, CND18 penetrates deepest, whereas CND19 remains closest to the surface. The polar nature of this group drives strong associations with water molecules and the phosphate groups of neighboring lipids, as confirmed by the hydrogen bonding analysis (Figure 5B). Among the tested analogs, CND15 (ethanolamine) and CND19 (piperazine) exhibit the highest exposure of their hydrophilic head groups to water, reinforcing their preference for polar interactions. Surprisingly, even CND16 and CND18—despite possessing highly hydrophobic head groups —are positioned in the membrane at a depth similar to cholesterol. This unexpected behavior is attributed to the strong electrostatic interactions facilitated by the protonated α-amino group, which effectively anchors these molecules at an elevated level within the bilayer. Notably, these interactions are significantly stronger than those observed for CND3, where the hydroxyl group of the serine linker provides weaker hydrogen bonding capabilities. These results show that both the head group and linker chemistry strongly affect how the molecules position themselves in the membrane and interact with other components. This highlights how CND analogs behave differently compared to cholesterol.

### 3.5. Live-Cell Imaging

**Figure 6.**
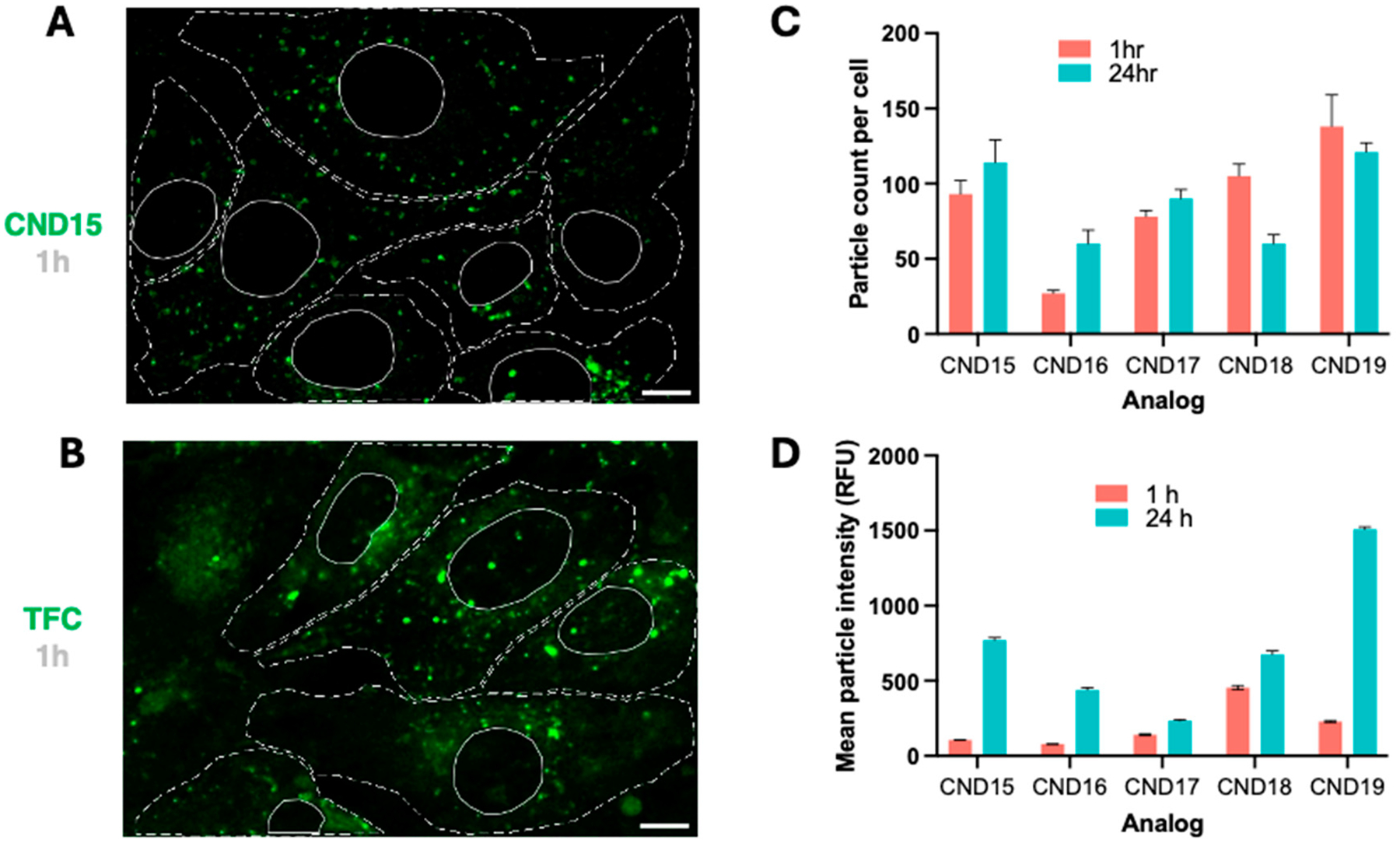
Intracellular distribution and quantification of CND fluorescence. (**A**) Typical distribution of the CND fluorescent signal after 1 h, showing characteristic green puncta for all tested CND analogs, although the intensity varies by analog. (**B**) Distribution of TFC after 1 h. Fluorescent puncta are observed, but the signal is more diffused. Dashed lines mark cell outlines, and solid lines mark nuclei, scale bar—10 μm. (**C**) Particle count per cell at 1 and 24 h for each analog, highlighting differences in the number of fluorescent puncta over time. (**D**) Mean particle intensity at 1 and 24 h, demonstrating that all CND analogs accumulate in vesicles, with the extent of accumulation influenced by the head group. Data are represented as mean ± SEM.

Live-cell imaging was performed using cultured 3T3 mouse fibroblasts. CND analogs and TFC were added directly to the cell media, and the cells were incubated for 1 h. TFC (also known as BODIPY-cholesterol or Bchol) is a well-established cholesterol analog widely used in cholesterol trafficking studies [6], and we have previously employed it as a reference in our prior study [11]. For colocalization studies, LysoView 650 was used as a lysosomal marker. After the 1-h incubation, cells were rinsed with dye-free media to remove unincorporated probes. Confocal imaging was conducted immediately following the incubation and again in a separate experiment 24 h later to assess the localization of the CNDs. At both the 1-h and 24-h time points, we observed a similar intracellular distribution of the dyes. Figure 6A presents the typical distribution of CND fluorescence, which is directly compared with the TFC pattern in Figure 6B. All tested CNDs produce distinct green puncta, though the intensity of these puncta varies with the analog used. In contrast, the TFC signal displays fewer puncta and a more diffuse fluorescence that surrounds the cell. This pattern is consistent with previous reports showing similar TFC behavior when introduced directly into cells from organic solvents [33]. Fluorescent CND puncta were used to generate regions of interest (ROIs) via machine learning, utilizing the Weka trainable segmentation tool. The mean intensity of each identified particle was quantified, and the particle count per cell was determined at each time point (Figure 6C). Overall, CNDs with a lysine linker exhibited higher cellular uptake than those with neutral linkers (CND1–CND10), likely due to the positively charged amino group on lysine. Among them, CND15 and CND19, which contain polar groups, showed the highest uptake at 1 h post-incubation, while CND16, with its relatively hydrophobic piperidine head group, exhibited the lowest. Interestingly, CND18, featuring a slightly less hydrophobic morpholine residue, was taken up much more efficiently than CND16.

At 24 h, the number of CND particles within cells remained constant or slightly increased for most analogs, except for CND18, whose puncta declined. This behavior mirrors that of CND6, which shares the same head group. Analysis of mean particle intensity changes over time (Figure 6D) indicated that all CND analogs accumulated in vesicles, with the extent of accumulation dependent on the head group. CND19, containing a piperazine moiety, showed the most pronounced fluorescence intensity increase over time, followed by ethanolamine-functionalized CND15. In contrast, CND17, featuring a hydroxyl group, exhibited the smallest increase. Focusing on particles between 0.38 and 1 μm (typical for lysosome size [34,35]), the largest fluorescence intensity increase was observed for CND15 (7.3-fold), followed by CND19 (6.6-fold) and CND16 (5.6-fold). The smallest changes occurred with CND18 (1.48-fold) and CND17 (1.6-fold). At 24 h post-incubation, CND19 produced the strongest fluorescence signal, twice as intense as the next best analog, CND15, a trend that was also evident at the 1-h mark. This strong fluorescence from CND19 is likely due to the pH-sensitive piperazine group, which, upon protonation in lysosomes, inhibits photoinduced electron transfer (PET) [29,36], thereby enhancing fluorescence.

Aside from variations in fluorescence intensity among the analogs, no distinct changes in fluorescence patterns are observed for CND15–CND19. Over time, however, fluorescent puncta tend to accumulate more in the perinuclear area. Our previous study showed that CNDs tend to accumulate in lysosomes and perhaps lipid droplets, depending on the CND structure [11]. Considering that the lipid droplet marker used in a previous study exhibited non-specific binding beyond lipid droplets [37], we opted not to use it in the current study. Instead, we exclusively used the lysosomal marker LysoView^™^ 650.

**Figure 7.**
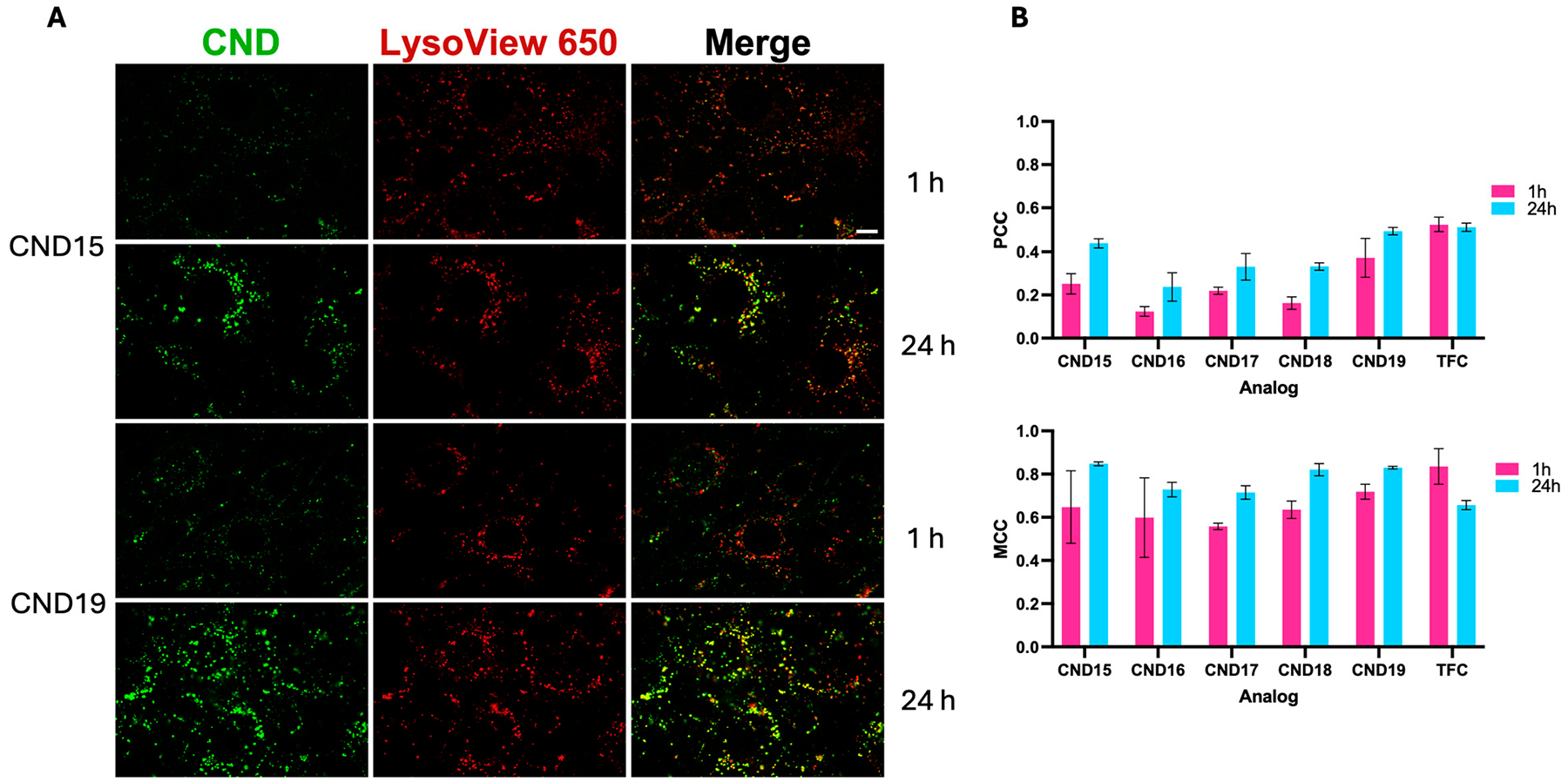
Colocalization of CND analogs with lysosomes at 1 and 24 h post-incubation. (**A**) Representative fluorescence images of CND (green) and LysoView 650 (red), with merged images (yellow) indicating colocalization. Shown here are examples for CND15 and CND19 at both time points. (**B**) Quantification of colocalization using Pearson’s correlation coefficient (PCC) and Manders’ overlap coefficient (MCC) for each CND analog, illustrating that CNDs progressively accumulate in lysosomes over time. TFC colocalization is shown as a reference. Data represented as means ± SEMs.

Colocalization analysis utilizes multiple metrics to comprehensively assess the spatial relationship between two markers. Pearson’s correlation coefficient (PCC) and Manders’ colocalization coefficient (MCC) are complementary, each offering distinct insights into colocalization [25]. PCC provides a measure of linear correlation between channels’ intensities, while MCC measures the fraction of overlap between two channels. The use of both methods provides a more complete understanding of colocalization. To evaluate colocalization among CNDs, TFC, and lysosomes, we used the EzColocalization plugin in ImageJ, applying Costes’ automatic thresholding method to ensure objective and reliable analysis. Colocalization analysis between CND analogs and lysosomes (LysoView) was assessed at 1 h and 24 h post-incubation (Figure 7). PCC values increased over time for all CNDs, consistent with a unidirectional trafficking of plasma membrane cholesterol into lysosomes. Among the tested analogs, CND19 exhibited the highest PCC values, followed by CND15, while CND16 showed the lowest colocalization, suggesting minimal lysosomal association. MCC values were consistently higher than PCC, indicating a significant fraction of CND fluorescence overlapped with lysosomes. At 24 h, MCC values exceeded 0.7 for all analogs, with CND15 and CND19 showing also the highest PCC (greater than 0.4), whereas at 1 h, CND16 and CND17 exhibited the lowest overlap. The increase in both PCC and MCC values over time suggests that CNDs are progressively transported into lysosomes, with CND19 demonstrating the strongest lysosomal retention, likely due to the influence of its piperazine head group.

Using TFC as a control, we evaluated its spatial overlap with LysoView (Figure 6B). TFC exhibited consistently high colocalization values at both 1 h and 24 h, as evidenced by its Pearson’s (PCC) and Manders’ (MCC) correlation coefficients, which were among the highest compared to the CND analogs. This strong colocalization is maintained over time, indicating that TFC strongly associates with lysosomes and progressively accumulates within them, in agreement with previous findings [11,33].

## 4. Discussion

The synthesized CND15–CND19 analogs were designed to investigate how L-lysine linker and distinct head groups influence their photophysical properties, lipid interactions, and cellular uptake, with the goal of evaluating their potential as fluorescent cholesterol probes. The incorporation of a lysine linker aimed to enhance hydrophilicity and cellular uptake through its positively charged α-amino group, potentially affecting lipid ordering. Our findings demonstrate that these structural modifications significantly influence the solvatochromic behavior, membrane partitioning, molecular orientation, and intracellular localization of these probes. The solvatochromic properties of CND15–CND19 revealed strong environmental sensitivity, a characteristic of 1,8-naphthalimide fluorophores. The observed red shifts in polar solvents, particularly in DMSO, indicate the presence of a charge transfer excited state, stabilized by solvent polarity [38]. Notably, CND15 exhibited the highest molar extinction coefficient and longest absorption wavelength, suggesting enhanced conjugation and stronger electronic transitions compared to the other analogs. In contrast, CND17 displayed the lowest extinction coefficient and a blue-shifted absorption maximum, indicative of weaker charge transfer interactions. Fluorescence measurements in octyl glucoside (OG) micelles versus water provided further insights into the probes’ aggregation tendencies. The higher fluorescence intensity of CND15, CND17, and CND19 in OG micelles suggests reduced self-quenching, whereas CND16 and CND18, which contain hydrophobic head groups, exhibited increased aggregation in aqueous environments. The pH-sensitive fluorescence of CND19 aligns with previous reports on piperazine-containing CND analogs, further confirming that the head group modulates the probe’s response to its environment [29,39]. Lipid phase partitioning studies using GUVs revealed distinct preferences among the analogs for liquid-ordered (Lo) and liquid-disordered (Ld) phases. CND17 and CND16 exhibited the strongest Lo phase affinity (~40%), while CND19, the most polar analog, displayed the lowest (~25%). These results indicate that both linker and head group polarity play a key role in Lo phase localization, reflecting earlier findings with cholesterol-based CND membrane probes. Furthermore, the partitioning behavior of these analogs correlates with their head group characteristics, underscoring the crucial contributions of linker hydrophobicity, hydrogen bonding capacity, and fluorophore orientation. Additionally, the positioning of the fluorophore along the linker plays a pivotal role in dictating phase partitioning preference.

Molecular dynamics simulations provided additional mechanistic insights into the behaviors of CND15–CND19 within lipid membranes. The calculated cholesterol tilt angles indicated significant deviations from native cholesterol, with CND16 and CND18 displaying the highest tilt angles, likely due to their hydrophobic head groups promoting deeper membrane embedding. The presence of the α-amino group influenced probe positioning within the bilayer, leading to increased interactions with lipid phosphate groups and water molecules. Surprisingly, even the hydrophobic analogs CND16 and CND18 were positioned higher in the membrane than cholesterol, suggesting that electrostatic interactions with the linker play a dominant role in membrane positioning. These results emphasize that both the head group and linker chemistry collectively determine how the probe integrates into lipid membranes.

Live-cell imaging revealed that all CND analogs localized intracellularly within vesicular structures, suggesting endosomal trafficking. The uptake efficiency varied significantly among the analogs, with CND15 and CND19 exhibiting the highest uptake at 1 h post-incubation, whereas CND16, with its hydrophobic piperidine head group, displayed the lowest uptake. Interestingly, CND18, containing a morpholine moiety, was taken up more efficiently than CND16, likely due to its slightly higher polarity. We have observed the same phenomenon with CND6 which was extremely well taken up by 3T3 fibroblasts, but not astrocytes. These findings highlight the impact of head group charge/polarity on cellular uptake efficiency.

Over 24 h, the number of intracellular CND particles remained stable or slightly increased, except for CND18, which exhibited a decline in fluorescence. This behavior mirrors that of CND6, which shares the same head group, suggesting a common mechanism influencing intracellular retention. Analysis of fluorescence intensity changes over time confirmed that all analogs accumulated in vesicles, with the extent of accumulation being head group dependent. CND19, featuring a piperazine head group, exhibited the most pronounced increase in fluorescence intensity, followed by ethanolamine-functionalized CND15. In contrast, CND17, with its hydroxyl head group, displayed the smallest increase. Notably, at 24 h, CND19 produced the strongest fluorescence signal, twice as intense as CND15, a trend also evident at 1 h. This enhanced fluorescence in CND19 is attributed to the pH-sensitive piperazine moiety, which, upon protonation inside the acidic lysosomes, inhibits photoinduced electron transfer (PET), thereby enhancing fluorescence.

Lysosomes play a crucial role in cholesterol metabolism and trafficking within cells [40]. Within the lysosome, acid lipases hydrolyze cholesterol esters to release free cholesterol [41]. This free cholesterol is then exported from the lysosome through the coordinated action of proteins like NPC1 and NPC2, allowing it to be redistributed to other cellular compartments such as the endoplasmic reticulum and plasma membrane [40]. In this sense, lysosomes act as a central hub for both the processing and redistribution of cholesterol, helping maintain cellular cholesterol homeostasis.

The colocalization analysis using Pearson’s correlation coefficient (PCC) and Manders’ colocalization coefficient (MCC) confirmed lysosomal accumulation of CNDs over time. PCC values increased across all analogs from 1 to 24 h, indicating progressive lysosomal localization. The highest PCC values were observed for CND19, followed by CND15, whereas CND16 displayed the lowest correlation, suggesting minimal lysosomal association. MCC values consistently exceeded PCC values, confirming substantial lysosomal colocalization. At 24 h, MCC values surpassed 0.7 for all analogs, with CND15 and CND19 showing the highest lysosomal retention. The progressive increase in both PCC and MCC values suggests that lysosomes play a key role in CND trafficking, with piperazine-containing CND19 exhibiting the strongest lysosomal association.

The performance of CND15–CND19 was compared to previously reported CND1–10 probes [11]. Although CND15 and CND19 exhibited best fluorescent signal intensity and cellular uptake among CND15–CND19 analogs, their preference for Lo partitioning was less optimal compared to CND3, which features a serine linker with a hydroxyl group at the beta position. These results suggest that the presence of an amino group in the alpha position of lysine introduces strain in the linker, affecting the interactions of the head group attached to the naphthalimide fluorophore. Consequently, this constrain leads to diminished Lo partitioning compared to probes featuring a serine linker. On the other hand, lysine-based analogs bearing a positively charged amino group accumulate in cells far more effectively than those bearing glycine or serine linkers published earlier [11]. We hypothesize that this enhanced accumulation arises from the increased positive charge, which promotes stronger interactions with phospholipids. These findings underscore the critical role of linker design in modulating the probe performance.

CND probes constitute a novel class of fluorescent cholesteryl probes built on a 1,8-naphthalimide (ND) scaffold, offering a modular design that allows flexible variation of linkers and head groups to fine-tune their properties. Compared with established probes such as NBD-cholesterol and BODIPY-modified cholesterol (Bchol) [6], CND probes feature substantially larger Stokes shifts (~130 nm vs. ~40 nm for NBD-cholesterol and ~10 nm for Bchol), making them particularly advantageous for co-imaging with other fluorescent reporters. In model membranes (GUVs), CND probes exhibit Lo/Ld partitioning ranging from 30 to 50%, with CND3 closely matching Bchol’s partitioning behavior. Notably, CND probes generally show stronger Lo domain partitioning than 22- or 25-NBD analogs. Furthermore, several members of this series (CND2–CND4, CND19) display pH-dependent fluorescence via a piperazine head group, providing additional versatility for imaging applications.

## 5. Conclusions

The results of this study further confirm that the head group and linker chemistry of CND analogs significantly influence their photophysical properties, membrane interactions, and intracellular localization. The ability of CND15–CND19 to partition into lipid domains and accumulate in lysosomes highlights their potential as versatile tools for studying cholesterol trafficking and membrane organization. Compared to existing cholesterol probes, these analogs offer unique advantages, including ease of cell uptake, enhanced environmental sensitivity, and tunable fluorescence properties. Future studies will explore alternative linker configurations to optimize the balance between fluorescent intensity, cellular uptake, and membrane partitioning, ultimately enhancing the utility of these fluorescent cholesterol analogs in biological applications.

## Supplementary Material

Supplementary material

**Supplementary Materials:** The following supporting information can be downloaded at: https://www.mdpi.com/article/10.3390/sci7020056/s1, Figure S1: The absorbance and emission spectra of CND15-CND19 analogs in various solvents.; Scheme S1: Synthetic scheme for CND15-CND19 analogs; Synthetic procedures and NMR/mass spectra.

## Figures and Tables

**Table 1.** Molar extinction coefficients (ε, M^−1^cm^−1^) for CND15–CND19 in chloroform and DMSO are presented along with the wavelengths at which each ε was measured.

Chloroform					
Analog	CND15	CND16	CND17	CND18	CND19
ε [M^−1^cm^−1^]	11,036	13,700	8282	10,510	10,920
λ_max_ [nm]	430	415	365	398	383
Dimethylsulfoxide					
Analog	CND15	CND16	CND17	CND18	CND19
ε [M^−1^cm^−1^]	12,911	10,028	5107	9961	10,544
λ_max_ [nm]	444	413	380	401	410

## Data Availability

The original contributions presented in this study are included in the article. Further inquiries can be directed to the corresponding author.
