# Supplementary material for: L-Lysine-Linked Modular Fluorescent Cholesteryl Mimics: Biophysical Properties, Molecular Interactions, and Cellular Applications
Source: Sci (Basel). Author manuscript; Available in PMC 2025 Sep 19. (PMC12445076; doi:10.3390/sci7020056)
Supplement: Supplementary material [file NIHMS2110195-supplement-Supplementary_material.pdf]

Supplementary Information for:

**L-Lysine-Linked Modular Fluorescent Cholesteryl Mimics: Biophysical Properties, Molecular Interactions, and Cellular Applications.**

Nicholas McInchak[1], Laura Stawikowska[1], Haylee Mesa[2], Jonathan Meade[2], Qi Zhang[1,2], Maciej J. Stawikowski[1]

[1] *Department of Chemistry and Biochemistry, Charles E. Schmidt College of Science, Florida Atlantic University, 777 Glades Rd., Boca Raton, FL 33431, USA*
[2] *Florida Atlantic Stiles-Nicholson Brain Institute, Florida Atlantic University, 5353 Parkside Dr., Jupiter, FL 33458, USA*

Correspondence should be addressed to
**Maciej J. Stawikowski** (mstawikowski@fau.edu)

## Table of Contents

| | PAGE |
|---|---|
| **Supplementary figures and tables referenced in the main text** | |
| Photophysical characterization of compounds | S3 |
| Chemical synthesis of CND15-CND19 probes | S4-S7 |
| **NMR and MS spectra of all intermediates and final compounds** | |
| NMR spectra | S8-S14 |
| MS spectra | S15-S18 |

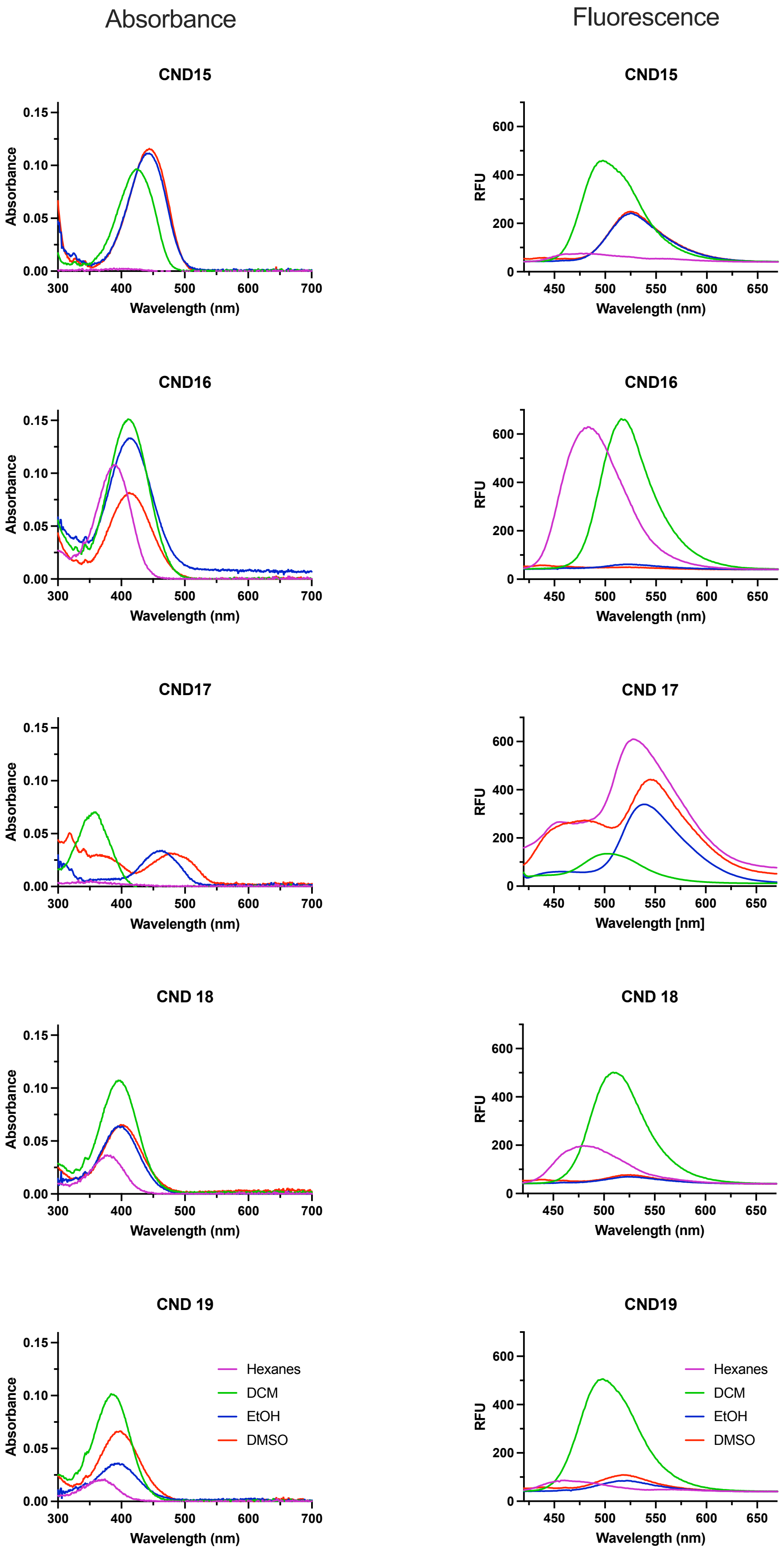
Absorbance
Fluorescence
CND15
CND15
CND16
CND16
CND17
CND 17
CND 18
CND 18
CND 19
CND19
Absorbance
RFU
Wavelength (nm)
Wavelength [nm]
Hexanes
DCM
EtOH
DMSO

**Fig. S1**. The absorbance and emission spectra of CND15-CND19 analogs in various solvents. Experimental conditions are described in the main manuscript in section 2.4 and 2.5.

## Chemical synthesis of CND15-CND19 probes

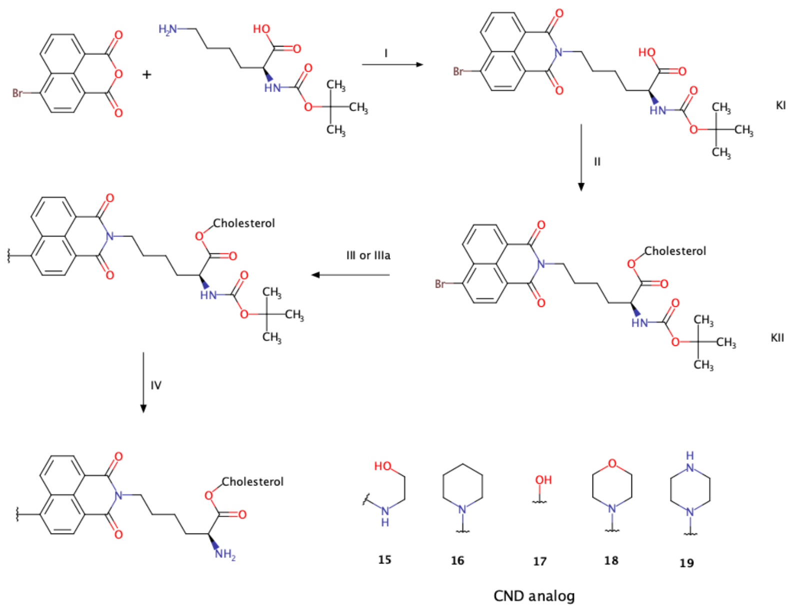


**Scheme S1.** Synthetic scheme for CND15-CND19 analogs.
(I) 4-bromo-1,8-naphthalic anhydride, Boc-Lys-OH, EtOH, reflux, (II) Cholesterol, DCC, DMAP, DCM, $N_2$, 0°C, (III) amine (except CND17) DMSO, 100 °C, (IIIa) N-hydroxyphthalimide, $K_2CO_3$, DMSO, 100 °C, then 10% HCl (IV) TFA, TIS, DCM, rt

## Synthesis of KI

In a round-bottom flask, 4-bromo-1,8-naphthalic anhydride (828 mg, 3.0 mmol) and Boc-lysine (886 mg, 3.6 mmol, 1.2 equiv.) were dissolved in 100 mL of ethanol, and diisopropylethylamine (523 µL, 3.6 mmol, 1.2 equiv.) was added. The reaction mixture was refluxed for 4 hours, evaporated to dryness, dissolved in ethyl acetate, diluted with water, and acidified. The organic layer was separated, dried over sodium sulfate, evaporated to dryness, and dried under vacuum. The crude orange-brown powder was purified on silica gel (20:1 chloroform:MeOH) to yield 990 mg of orange powder (65% yield). $^1$H-NMR (400 MHz, $CDCl_3$): δ 8.64 (d, J = 7.2 Hz, 1H), 8.55 (d, J = 8.5 Hz, 1H), 8.39 (d, J = 7.9 Hz, 1H), 8.02 (d, J = 7.8 Hz, 1H), 7.83 (t, J = 7.9 Hz, 1H), 5.30 (d, J = 8.0 Hz, 1H), 4.32 (d, J = 8.5 Hz, 1H), 4.27–4.11 (m, 2H), 3.66 (s, 1H), 1.98 (s, 1H), 1.81 (s, 3H), 1.45 (s, 9H). $^{13}$C-NMR (400 MHz, $CDCl_3$): δ 176.76, 163.58, 155.84, 134.20, 133.20, 132.08, 131.24, 131.03, 130.38, 130.29, 128.74, 128.59, 128.02, 122.80, 121.94, 81.47, 80.05, 77.41, 77.29, 77.09, 76.77, 70.50, 53.39, 39.95, 31.81, 29.71, 28.34, 27.54, 22.75. MALDI-TOF: $[M+Na]^+$ calcd. m/z = 527.0788 Da, obs. m/z = 526.6383 Da.

## Synthesis of KII

In a round-bottom flask, KI (990 mg, 1.97 mmol), cholesterol (759 mg, 1.97 mmol, 1 equiv.), DCC (486 mg, 2.36 mmol, 1.2 equiv.), and DMAP (122 mg, 1.96 mmol, 1 equiv.) were dissolved in 20 mL of anhydrous chloroform, stirred over ice, and allowed to proceed for 24 hours. The reaction mixture was evaporated to dryness, dissolved in ethyl acetate, and filtered to remove the dicyclohexylurea byproduct. The organic layer was washed with water and brine before being evaporated to

dryness. The crude product was purified on silica gel (3:1 hexanes:EtOAc) to yield 893 mg of white powder (60% yield). $^{1}$H-NMR (400 MHz, $CDCl_3$): δ 8.66 (dd, J = 7.3, 1.2 Hz, 1H), 8.57 (dd, J = 8.4, 1.2 Hz, 1H), 8.42 (d, J = 7.8 Hz, 1H), 8.04 (d, J = 7.9 Hz, 1H), 7.85 (dd, J = 8.5, 7.3 Hz, 1H), 5.17 (dd, J = 16.3, 6.5 Hz, 2H), 4.68–4.57 (m, 1H), 4.29–4.09 (m, 4H), 2.22 (d, J = 8.1 Hz, 2H), 2.06 (s, 3H), 1.99–1.69 (m, 11H), 1.69–1.48 (m, 9H), 1.44 (s, 12H), 1.40–1.31 (m, 5H), 1.31–1.27 (m, 7H), 1.27–1.15 (m, 6H), 1.15–1.05 (m, 6H), 1.05–0.87 (m, 21H), 0.87–0.80 (m, 4H), 0.70 (s, 3H). $^{13}$C-NMR (400 MHz, $CDCl_3$): δ 172.16, 163.66, 155.40, 139.24, 133.28, 132.13, 131.31, 131.12, 130.63, 130.30, 128.99, 128.10, 123.02, 122.76, 122.15, 79.65, 77.36, 77.25, 77.05, 76.73, 74.92, 60.42, 56.66, 56.12, 53.50, 49.97, 42.30, 40.03, 39.71, 39.52, 37.91, 36.90, 36.64, 36.51, 36.19, 36.08, 35.80, 34.67, 34.53, 32.50, 31.88, 31.80, 31.61, 29.07, 28.34, 28.24, 28.03, 27.73, 27.66, 25.28, 24.70, 24.31, 23.83, 23.37, 22.84, 22.71, 22.67, 22.58, 21.06, 21.01, 20.71, 19.30, 18.77, 18.72, 14.21, 14.14, 11.87, 11.45. MALDI-TOF: $[M+Na]^+$ calcd. m/z = 897.4223 Da, obs. m/z = 896.6554 Da.

**Synthesis of CND15**

KII (200 mg, 0.23 mmol) was dissolved in 10 mL of DMSO and stirred at 100°C until fully solubilized. Ethanolamine (55 µL, 0.92 mmol, 8 equiv.) was added, and the reaction mixture was stirred for 1.5 hours before being diluted with 100 mL of water and acidified with 10% HCl (v/v). The aqueous layer was extracted with EtOAc (3 × 25 mL), and the organic layer was dried over sodium sulfate and evaporated to dryness. The crude solid was dissolved in 3 mL DCM, and trifluoroacetic acid (3 mL) and triisopropylsilane (140 µL) were added. The reaction was stirred for 1 hour, neutralized to pH 10 with sodium carbonate, and the aqueous layer was extracted with DCM (3 × 25 mL). The organic layer was dried and purified on TEA-deactivated silica gel (20:1 DCM:MeOH) to yield 58 mg of bright orange powder (33% yield). $^{1}$H-NMR (400 MHz, $CDCl_3$): δ 8.25 (d, J = 7.3 Hz, 1H), 8.17 (d, J = 8.4 Hz, 1H), 8.02 (d, J = 8.5 Hz, 1H), 7.33 (t, J = 7.8 Hz, 1H), 6.48 (d, J = 8.6 Hz, 1H), 6.14 (d, J = 5.1 Hz, 1H), 5.26 (d, J = 4.8 Hz, 1H), 4.60 (tdd, J = 10.6, 6.6, 3.9 Hz, 1H), 4.08 (t, J = 7.3 Hz, 2H), 4.01 (t, J = 5.1 Hz, 2H), 3.46 (h, J = 5.2 Hz, 3H), 2.81 (s, 1H), 2.32–2.22 (m, 2H), 2.01 (s, 3H), 1.86–1.61 (m, 8H), 1.61–1.30 (m, 14H), 1.25 (t, J = 4.6 Hz, 2H), 1.10 (dddt, J = 26.6, 16.9, 13.7, 6.0 Hz, 9H), 0.96 (s, 4H), 0.95–0.83 (m, 13H), 0.66 (s, 3H). $^{13}$C-NMR (400 MHz, $CDCl_3$): δ 175.30, 164.45, 164.07, 149.79, 139.27, 134.25, 130.90, 129.34, 126.36, 124.35, 122.80, 122.27, 120.08, 109.51, 104.17, 77.41, 77.29, 77.09, 76.77, 74.66, 59.89, 56.63, 56.11, 54.23, 49.95, 45.64, 42.28, 39.78, 39.68, 39.51, 37.98, 36.90, 36.51, 36.18, 35.80, 34.49, 31.86, 31.78, 29.71, 28.24, 28.02, 27.86, 27.71, 24.28, 23.85, 23.20, 22.85, 22.59, 21.00, 19.29, 18.72, 11.86. MALDI-TOF: $[M+Na]^+$ calcd. m/z = 776.4972 Da, obs. m/z = 775.9271 Da.

**Synthesis of CND16**

KII (200 mg, 0.23 mmol) was dissolved in 10 mL of DMSO and stirred at 100°C until fully solubilized. Piperidine (226 µL, 0.46 mmol, 10 equiv.) was added, and the reaction was stirred for 1.5 hours. The reaction mixture was diluted with water and brine (100 mL, 1:1) and acidified with 10% HCl (v/v). The aqueous layer was extracted with DCM (3 × 25 mL), and the organic layer was dried over sodium sulfate and evaporated to dryness. The crude solid was dissolved in 3 mL DCM, and trifluoroacetic acid (3 mL) and triisopropylsilane (140 µL) were added. The reaction was stirred for 1 hour, neutralized to pH 9 with sodium carbonate, and the aqueous layer was extracted with DCM (3 × 25 mL). The organic layer was dried and purified on TEA-deactivated silica gel (50:1 DCM:MeOH) to yield 91 mg of bright orange powder (51% yield). $^{1}$H-NMR (400 MHz, $CDCl_3$): δ 8.51 (dd, J = 7.3, 1.2 Hz, 1H), 8.43 (d, J = 8.1 Hz, 1H), 8.33 (dd, J = 8.4, 1.2 Hz, 1H), 7.62 (dd, J = 8.5, 7.3 Hz, 1H), 7.12 (d, J = 8.2 Hz, 1H), 5.26–5.20 (m, 1H), 4.59 (dtd, J = 12.8, 7.6, 4.0 Hz, 1H), 4.19–4.11 (m, 2H), 3.40 (dd, J = 7.2, 5.5 Hz, 1H), 3.19 (t, J = 5.3 Hz, 4H), 2.30–2.20 (m, 2H), 1.95 (tt, J = 15.7, 4.2 Hz, 2H), 1.86 (p, J = 5.5 Hz, 5H), 1.80 (dt, J = 5.9, 2.6 Hz, 3H), 1.76 (dd, J = 11.5, 4.9 Hz, 3H), 1.71 (dt, J = 11.2, 5.6 Hz, 3H), 1.66 (s, 17H), 1.21–1.02 (m, 8H), 0.95 (s, 6H), 0.90–0.81 (m, 10H), 0.65 (s, 3H). $^{13}$C-NMR (400 MHz, $CDCl_3$): δ 175.49, 164.48, 163.99,

157.20, 139.38, 132.63, 130.96, 130.51, 129.85, 126.19, 125.32, 123.05, 122.63, 115.87, 114.65, 77.46, 77.34, 77.14, 76.82, 74.32, 56.63, 56.09, 54.53, 54.45, 49.95, 42.26, 39.91, 39.69, 39.50, 37.98, 36.93, 36.49, 36.16, 35.78, 34.89, 31.85, 31.79, 28.23, 28.00, 27.97, 27.71, 26.23, 24.35, 24.28, 23.83, 23.26, 22.85, 22.59, 21.00, 19.29, 18.72, 11.85. MALDI-TOF: $[M+Na]^+$ calcd. m/z = 800.5336 Da, obs. m/z = 799.9279 Da.

**Synthesis of CND17**

KII (200 mg, 0.23 mmol), N-hydroxyphthalimide (245 mg, 1.15 mmol, 5 equiv.), and potassium carbonate (159 mg, 1.15 mmol, 5 equiv.) were dissolved in 10 mL of DMSO and stirred at 100°C for 1.5 hours. The reaction mixture was diluted with 50 mL of water and acidified with 10% HCl (v/v). The aqueous layer was extracted with EtOAc (3 × 25 mL), and the organic layer was dried over sodium sulfate and evaporated to dryness. The crude product was dissolved in 3 mL DCM, and trifluoroacetic acid (3 mL) and triisopropylsilane (140 µL) were added. The reaction was stirred for 1 hour, neutralized to pH 7–8 with sodium carbonate, and the aqueous layer was extracted with DCM (5 × 20 mL). The organic layer was dried and purified on a Buchi Pure Flash system using a 25g ecoflex column (2–5% MeOH in DCM gradient over 30 CVs) to yield 100 mg of orange powder (61% yield). $^1$H-NMR (400 MHz, $CDCl_3$): δ 8.40 (dd, J = 7.3, 1.2 Hz, 1H), 8.28 (dd, J = 8.3, 1.3 Hz, 1H), 8.19 (d, J = 8.2 Hz, 1H), 7.46–7.38 (m, 1H), 6.79 (d, J = 8.2 Hz, 1H), 5.35–5.29 (m, 1H), 4.72–4.61 (m, 2H), 4.56 (s, 1H), 4.25–4.09 (m, 2H), 3.58 (dd, J = 7.9, 4.5 Hz, 1H), 2.69 (s, 2H), 2.30 (d, J = 8.1 Hz, 2H), 2.08–1.83 (m, 7H), 1.83 (s, 2H), 1.64–1.56 (m, 4H), 1.56–1.32 (m, 9H), 1.27 (t, J = 5.4 Hz, 2H), 1.22–1.04 (m, 8H), 1.00 (d, J = 8.9 Hz, 6H), 0.95–0.84 (m, 11H), 0.68 (s, 3H). $^{13}$C-NMR (400 MHz, $CDCl_3$): δ 174.40, 164.93, 164.53, 161.31, 139.18, 133.87, 131.42, 130.95, 129.69, 129.34, 128.86, 124.85, 122.98, 121.74, 112.40, 109.78, 77.36, 77.24, 77.04, 76.72, 75.18, 56.67, 56.16, 53.85, 49.99, 42.31, 40.77, 39.72, 39.53, 39.36, 37.99, 36.91, 36.54, 36.20, 35.81, 33.66, 31.88, 31.81, 29.71, 28.24, 28.02, 27.71, 24.29, 23.87, 22.83, 22.79, 22.58, 21.02, 19.29, 19.17, 18.73, 11.86, 0.01. MALDI-TOF: $[M+Na]^+$ calcd. m/z = 733.4550 Da, obs. m/z = 732.9801 Da.

**Synthesis of CND18**

KII (200 mg, 0.23 mmol) was dissolved in 10 mL of DMSO and stirred at 100°C until fully solubilized. Morpholine (400 µL, 4.6 mmol, 20 equiv.) was added, and the reaction was stirred for 1.5 hours. The reaction was diluted with 100 mL of water and acidified with 10% HCl (v/v). The aqueous layer was extracted with EtOAc (3 × 25 mL), and the organic layer was dried over sodium sulfate and evaporated to dryness. The crude solid was dissolved in 3 mL DCM, and trifluoroacetic acid (3 mL) and triisopropylsilane (140 µL) were added. The reaction was stirred for 1 hour, neutralized to pH 10 with sodium carbonate, and the aqueous layer was extracted with DCM (3 × 25 mL). The organic layer was dried and purified on TEA-deactivated silica gel (100:1 DCM:MeOH) to yield 117 mg of bright yellow powder (72% yield). $^1$H-NMR (400 MHz, $CDCl_3$): δ 8.58 (dd, J = 7.2, 1.2 Hz, 1H), 8.52 (d, J = 8.1 Hz, 1H), 8.42 (dd, J = 8.6, 1.2 Hz, 1H), 7.71 (dd, J = 8.5, 7.2 Hz, 1H), 7.23 (d, J = 8.1 Hz, 1H), 5.27 (dd, J = 5.0, 2.0 Hz, 1H), 4.64 (s, 1H), 4.19 (dd, J = 8.2, 6.6 Hz, 2H), 4.03 (dd, J = 5.8, 3.3 Hz, 4H), 3.45–3.37 (m, 1H), 3.30–3.24 (m, 4H), 2.33–2.23 (m, 2H), 2.06–1.89 (m, 3H), 1.89–1.72 (m, 7H), 1.64 (s, 7H), 1.51 (dd, J = 15.5, 9.6 Hz, 4H), 1.47–1.32 (m, 6H), 1.32–1.17 (m, 3H), 1.17–0.94 (m, 13H), 0.94–0.87 (m, 8H), 0.86 (d, J = 1.9 Hz, 3H), 0.68 (s, 3H). $^{13}$C-NMR (400 MHz, $CDCl_3$): δ 175.56, 164.37, 163.92, 155.57, 139.41, 132.53, 131.18, 130.02, 129.84, 126.10, 125.85, 123.29, 122.67, 117.14, 114.93, 77.41, 77.30, 77.10, 76.78, 74.36, 66.99, 56.64, 56.09, 54.48, 53.45, 49.96, 42.29, 40.02, 39.70, 39.51, 38.00, 36.93, 36.53, 36.17, 35.80, 34.88, 31.88, 31.82, 28.24, 28.02, 27.95, 27.73, 24.32, 23.83, 23.28, 22.86, 22.60, 21.01, 19.32, 18.73, 11.87. MALDI-TOF: $[[M+Na]^+$ calcd. m/z = 802.5129 Da, obs. m/z = 801.9852 Da.

**Synthesis of CND19**

KII (100 mg, 0.23 mmol) and piperazine (198 mg, 2.30 mmol, 10 equiv.) were dissolved in 10 mL of DMSO and stirred at 100°C for 1.5 hours. The reaction was diluted with 100 mL of water and acidified with 10% HCl (v/v). The aqueous layer was extracted with EtOAc (3 × 25 mL), and the organic layer was dried over sodium sulfate and evaporated to dryness. The crude solid was dissolved in 10 mL DCM, and trifluoroacetic acid (2 mL) and triisopropylsilane (30 μL) were added. The reaction was stirred for 1hour, neutralized to pH 7 with sodium carbonate, and the aqueous layer was extracted with DCM (3 × 25 mL). The organic layer was dried and purified using a Buchi Pure Flash system with a 12g ecoflex column. The combined fractions were evaporated to dryness to yield 96 mg of orange powder (59% yield). $^1$H-NMR (400 MHz, $CDCl_3$): δ 8.57 (dd, J = 7.3, 1.2 Hz, 1H), 8.51 (d, J = 8.0 Hz, 1H), 8.42 (dd, J = 8.5, 1.2 Hz, 1H), 7.69 (dd, J = 8.5, 7.2 Hz, 1H), 7.21 (d, J = 8.1 Hz, 1H), 5.32–5.24 (m, 1H), 4.60 (dd, J = 10.9, 6.2 Hz, 1H), 4.18 (t, J = 7.4 Hz, 2H), 3.49 (s, 2H), 3.40 (dd, J = 7.2, 5.5 Hz, 1H), 3.28–3.16 (m, 7H), 2.26 (d, J = 7.2 Hz, 2H), 2.05–1.90 (m, 3H), 1.84 (d, J = 3.2 Hz, 2H), 1.82–1.71 (m, 7H), 1.69–1.31 (m, 16H), 1.30–1.24 (m, 2H), 1.24–1.11 (m, 4H), 1.11–1.02 (m, 4H), 1.02–0.83 (m, 15H), 0.68 (s, 3H). $^{13}$C-NMR (400 MHz, CDCl3): δ 175.54, 164.49, 164.02, 156.31, 139.40, 132.63, 131.13, 130.29, 129.87, 126.15, 125.66, 123.19, 122.69, 116.65, 114.96, 77.41, 77.29, 77.09, 76.77, 74.41, 56.65, 56.09, 54.44, 54.28, 49.96, 46.14, 42.29, 39.99, 39.70, 39.51, 38.00, 36.93, 36.53, 36.17, 35.80, 34.82, 31.87, 31.81, 28.24, 28.02, 27.94, 27.72, 24.31, 23.82, 23.27, 22.86, 22.59, 21.01, 19.32, 18.72, 11.87. MALDI-TOF: $[M+Na]^+$ calcd. m/z = 801.528925 Da, obs. m/z = 800.9048 Da.

KI

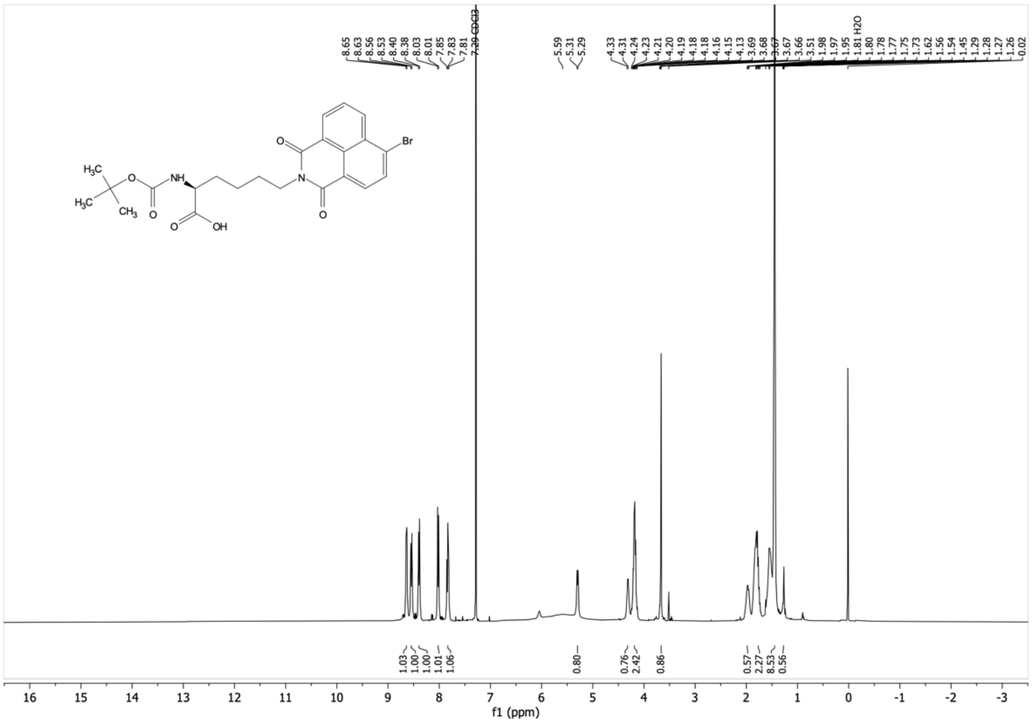
8.65
8.63
8.56
8.53
8.40
8.38
8.03
8.01
7.85
7.83
7.81
7.29 CDCl3
5.59
5.31
5.29
4.33
4.31
4.24
4.23
4.21
4.20
4.19
4.18
4.18
4.16
4.15
4.13
3.69
3.68
3.67
3.66
3.51
1.98
1.97
1.95
1.81 H2O
1.80
1.78
1.77
1.75
1.73
1.62
1.56
1.54
1.45
1.29
1.28
1.27
1.26
0.02
Br
O
N
H3C
H3C
CH3
NH
OH
1.03
1.00
1.00
1.01
1.06
0.80
0.76
2.42
0.86
0.57
2.27
8.53
0.56
f1 (ppm)

KI

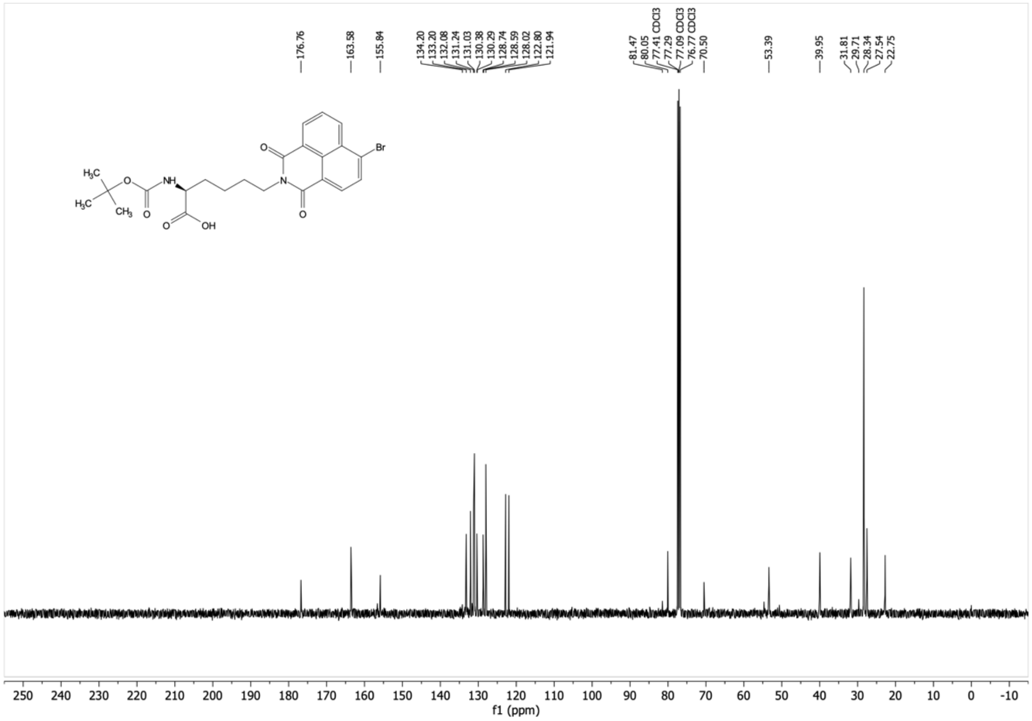
176.76
163.58
155.84
134.20
133.20
132.08
131.24
131.03
130.38
130.29
128.74
128.59
128.02
122.80
121.94
81.47
80.05
77.41 CDCl3
77.29
77.09 CDCl3
76.77 CDCl3
70.50
53.39
39.95
31.81
29.71
28.34
27.54
22.75
Br
O
N
H3C
H3C
CH3
NH
OH
f1 (ppm)

KII

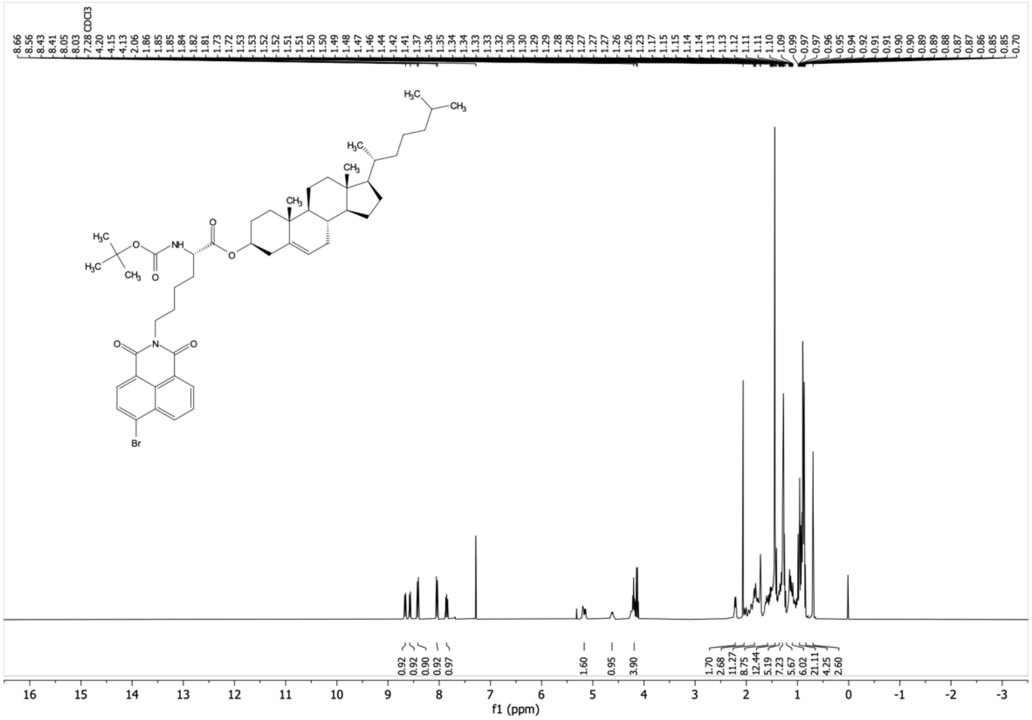
8.66 8.56 8.43 8.41 8.05 8.03 7.28 CDCl3 4.20 4.15 4.13 2.06 1.86 1.85 1.85 1.84 1.82 1.81 1.73 1.72 1.53 1.53 1.52 1.52 1.51 1.51 1.50 1.50 1.49 1.48 1.47 1.46 1.44 1.42 1.41 1.37 1.36 1.35 1.34 1.34 1.33 1.33 1.32 1.30 1.30 1.29 1.29 1.28 1.28 1.27 1.27 1.27 1.26 1.26 1.23 1.17 1.15 1.15 1.14 1.14 1.13 1.13 1.12 1.11 1.11 1.11 1.10 1.09 0.99 0.97 0.97 0.96 0.95 0.94 0.92 0.91 0.91 0.90 0.90 0.90 0.89 0.89 0.88 0.87 0.87 0.86 0.85 0.85 0.70
0.92 0.92 0.90 0.92 0.97 1.60 0.95 3.90 1.70 2.68 11.27 8.75 12.44 5.19 7.23 5.67 6.02 21.11 4.25 2.60
f1 (ppm)
NH
O
N
Br

KII

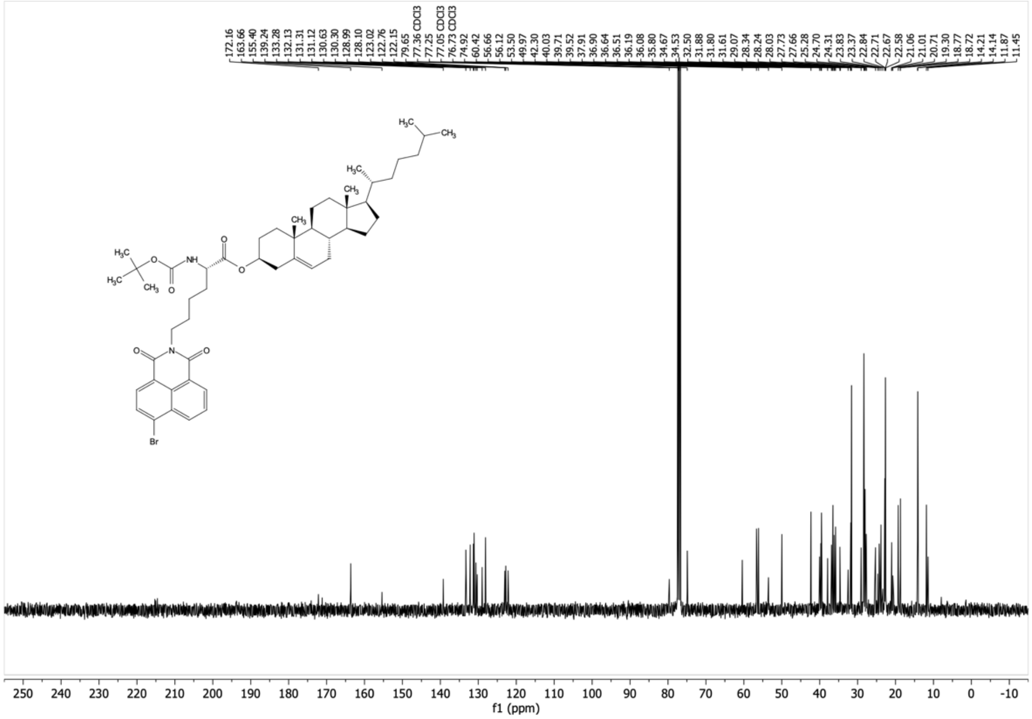
172.16 163.66 155.40 139.24 133.28 132.13 131.31 131.12 130.63 130.30 128.99 128.10 123.02 122.76 122.15 79.65 77.36 CDCl3 77.25 77.05 CDCl3 76.73 CDCl3 74.92 60.42 56.66 56.12 53.50 49.97 42.30 40.03 39.71 39.52 37.91 36.90 36.64 36.51 36.19 36.08 35.80 34.67 34.53 32.50 31.88 31.80 31.61 29.07 28.34 28.24 28.03 27.73 27.66 25.28 24.70 24.31 23.83 23.37 22.84 22.71 22.67 22.58 21.06 21.01 20.71 19.30 18.77 18.72 14.21 14.14 11.87 11.45
f1 (ppm)
NH
O
N
Br

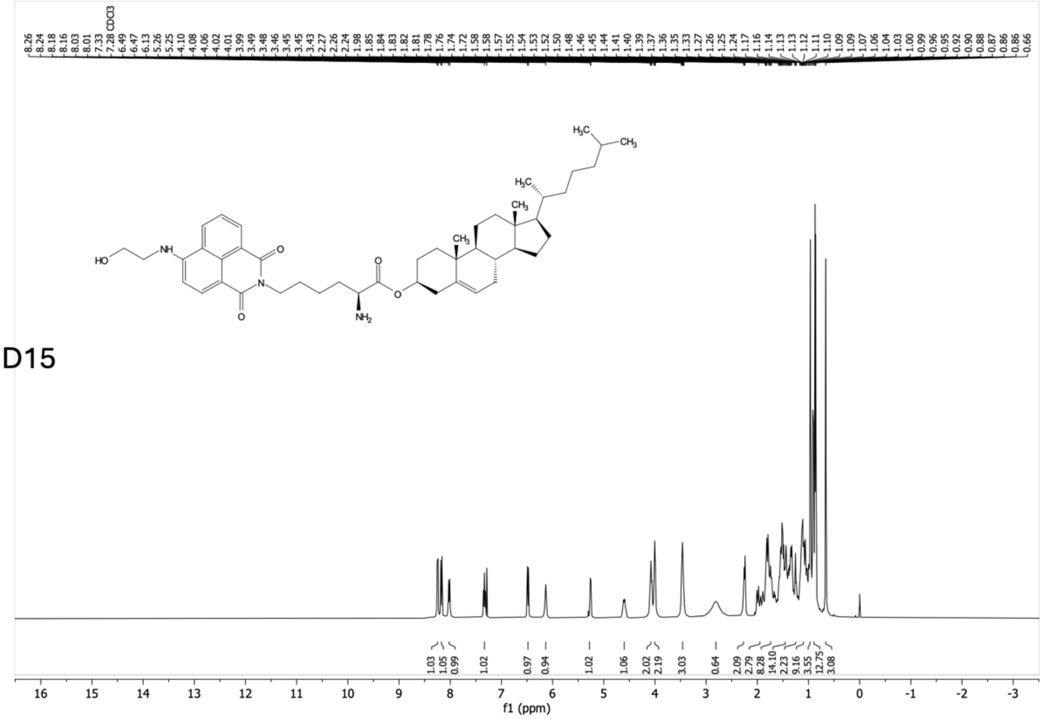


CND15

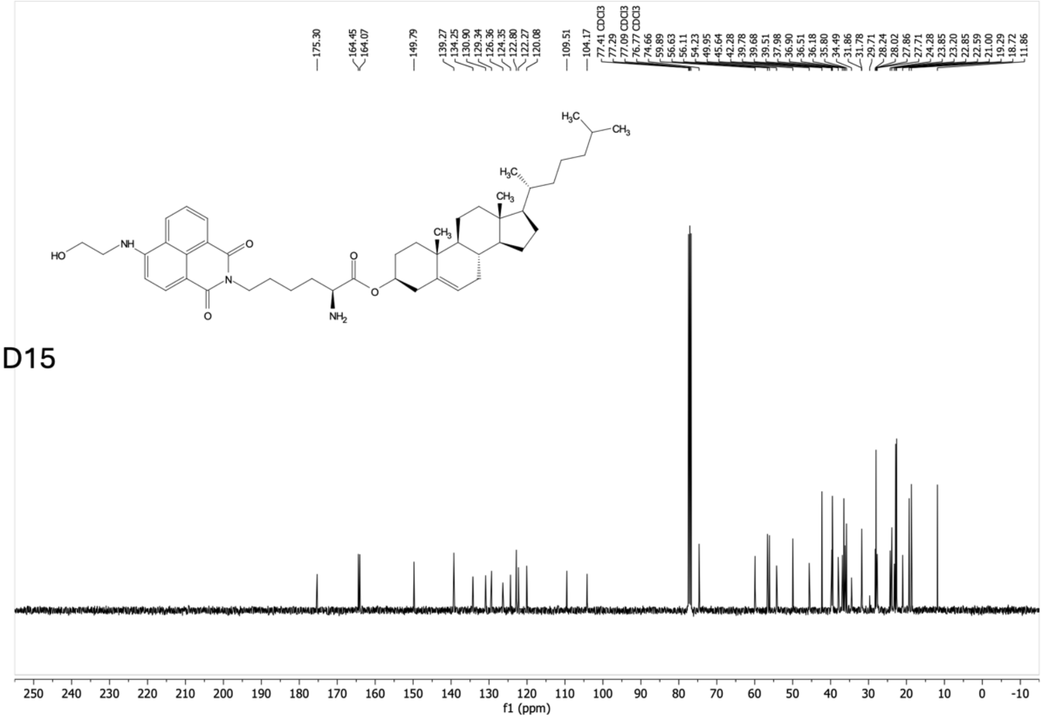


CND15

CND16

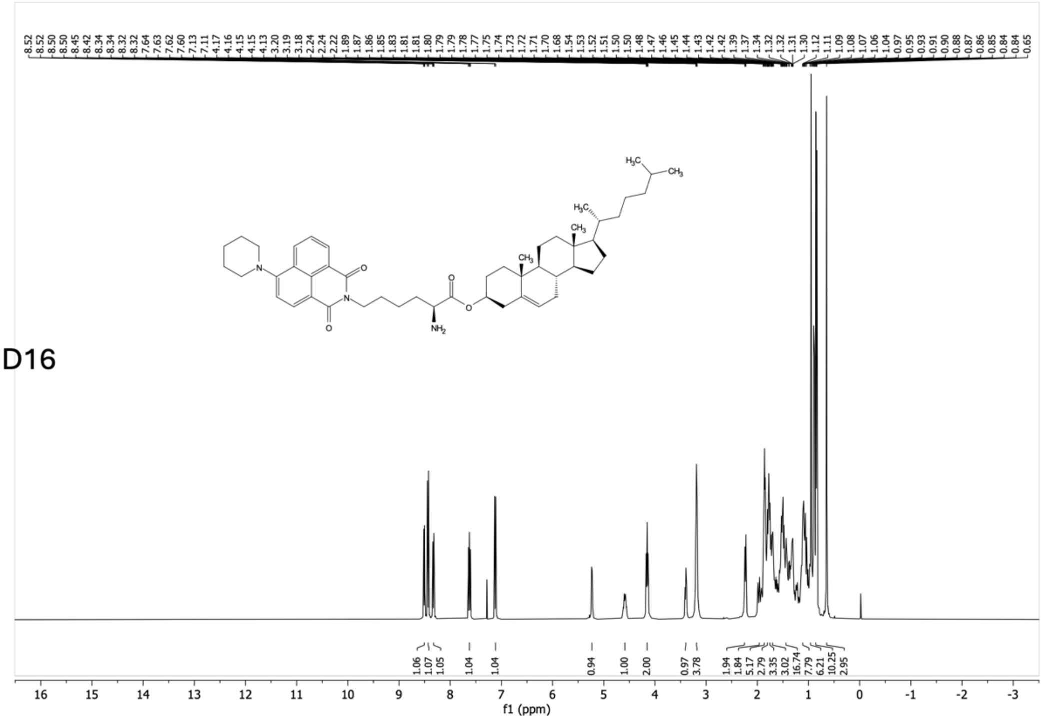


CND16

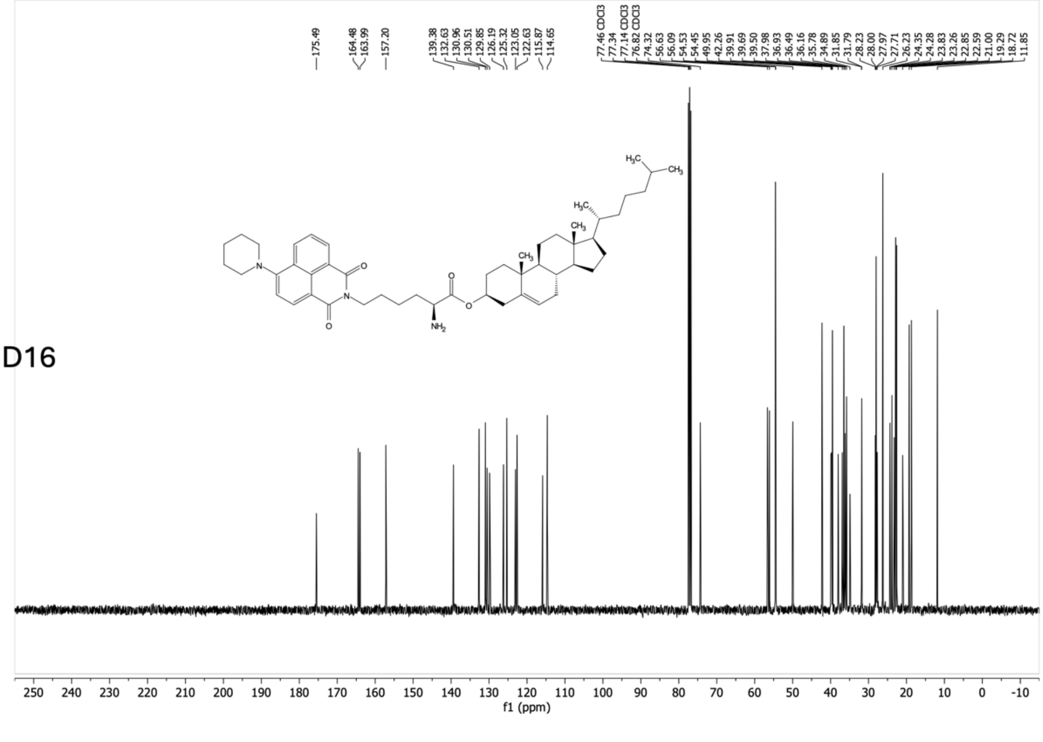

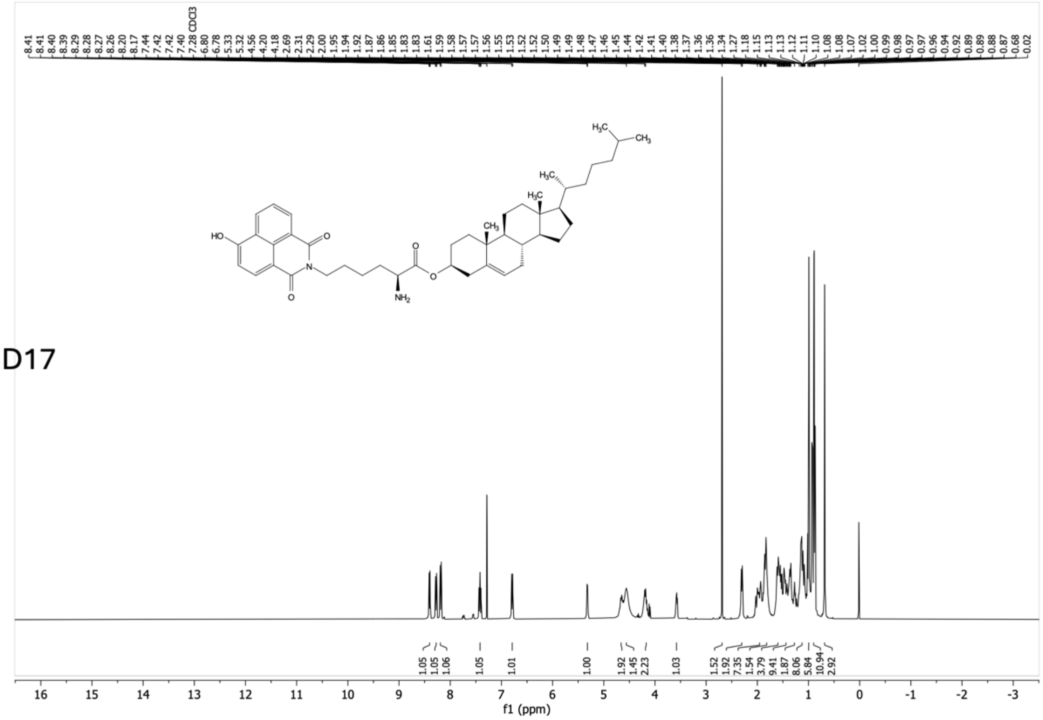


CND17

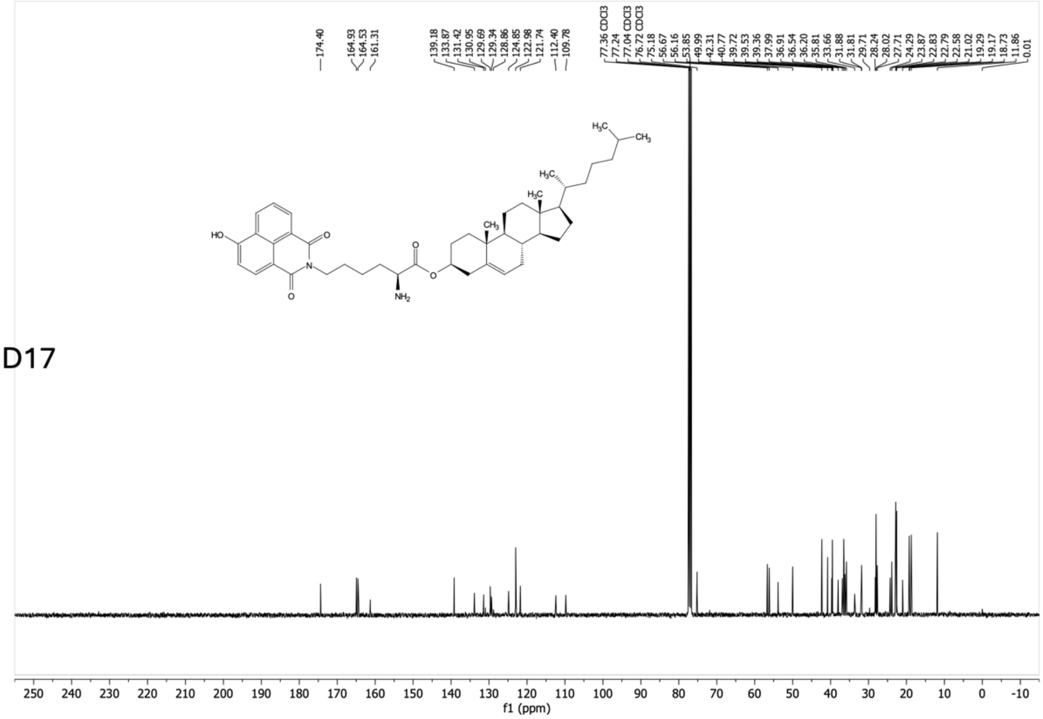


CND17

CND18

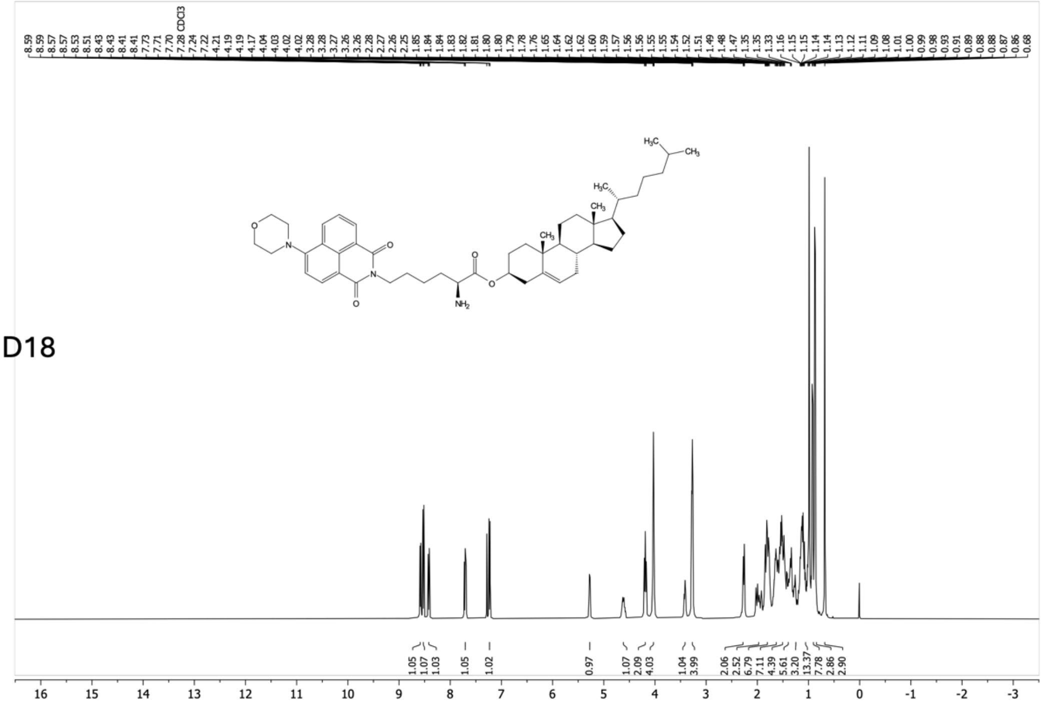
8.59 8.59 8.57 8.57 8.53 8.51 8.43 8.43 8.41 8.41 7.73 7.71 7.70 CDCl3 7.28 7.24 7.22 4.21 4.19 4.19 4.17 4.04 4.03 4.02 4.02 3.28 3.28 3.27 3.26 2.28 2.27 2.26 2.25 1.85 1.84 1.84 1.83 1.82 1.81 1.80 1.80 1.79 1.78 1.76 1.65 1.64 1.62 1.62 1.60 1.59 1.57 1.56 1.56 1.55 1.55 1.54 1.52 1.51 1.49 1.48 1.47 1.35 1.35 1.33 1.16 1.15 1.15 1.14 1.14 1.13 1.12 1.11 1.09 1.08 1.01 1.00 0.99 0.98 0.93 0.91 0.89 0.88 0.88 0.87 0.86 0.68
1.05 1.07 1.03 1.05 1.02 0.97 1.07 2.09 4.03 1.04 3.99 2.06 2.52 6.79 7.11 4.39 5.61 3.20 13.37 7.78 2.86 2.90
16 15 14 13 12 11 10 9 8 7 6 5 4 3 2 1 0 -1 -2 -3

CND18

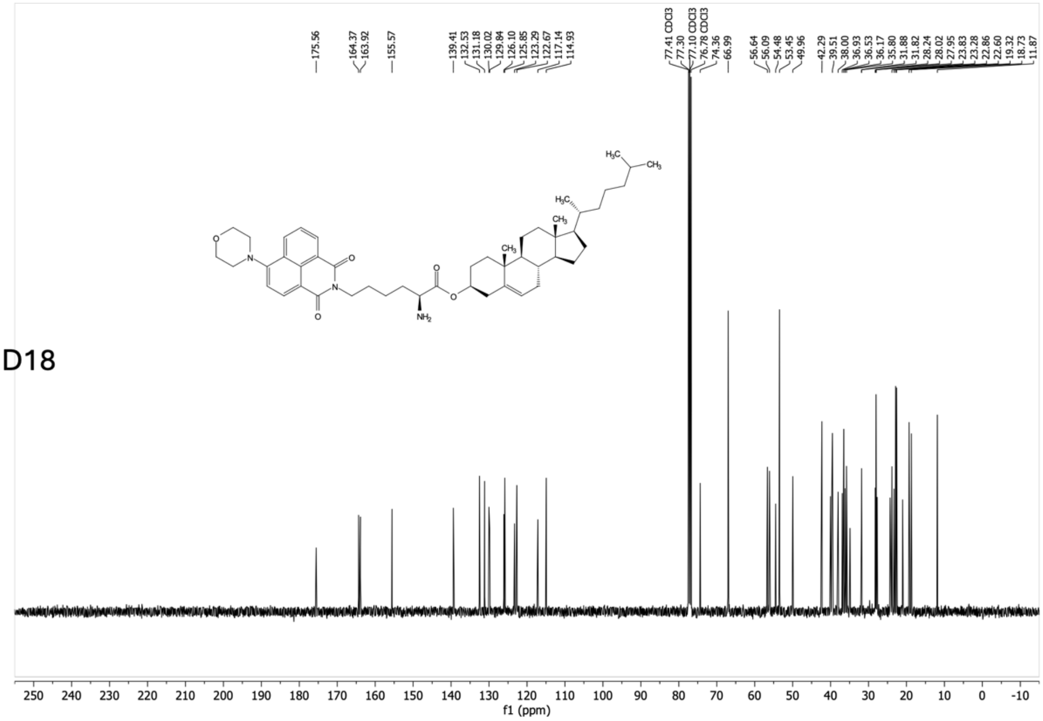
175.56 164.37 163.92 155.57 139.41 132.53 131.18 130.02 129.84 126.10 125.85 123.29 122.67 117.14 114.93 77.41 CDCl3 77.30 77.10 CDCl3 76.78 CDCl3 74.36 66.99 56.64 56.09 54.48 53.45 49.96 42.29 39.51 38.00 36.93 36.53 36.17 35.80 31.88 31.82 28.24 28.02 27.95 23.83 23.28 22.86 22.60 19.32 18.73 11.87
250 240 230 220 210 200 190 180 170 160 150 140 130 120 110 100 90 80 70 60 50 40 30 20 10 0 -10
f1 (ppm)

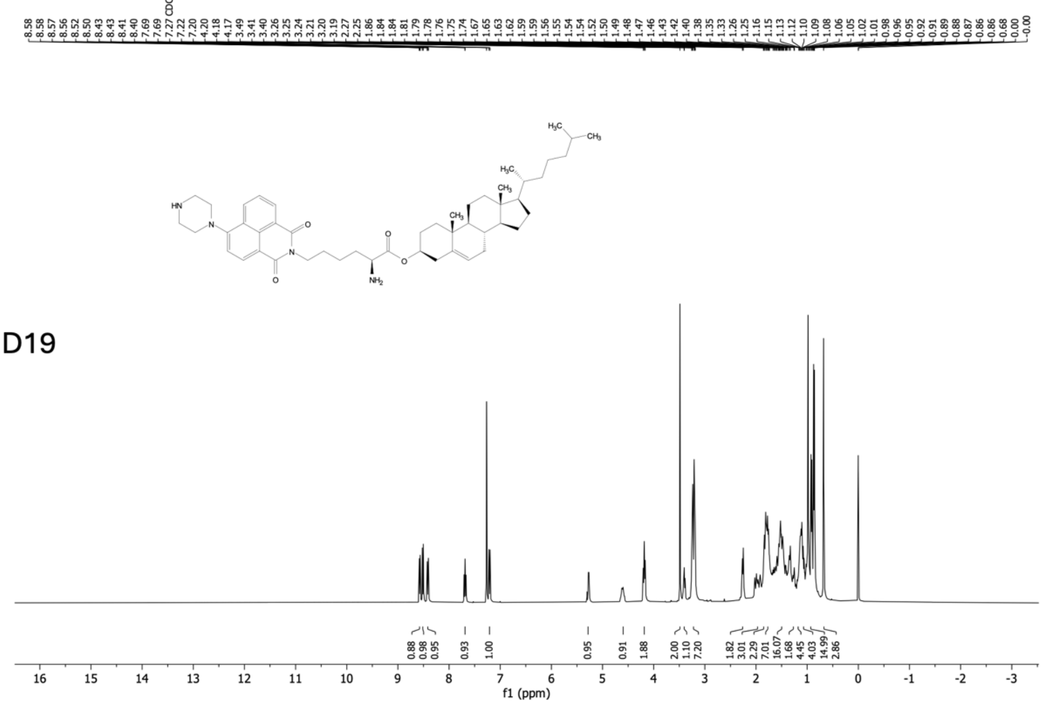
8.58 8.58 8.57 8.56 8.52 8.50 8.43 8.43 8.41 8.40 7.69 7.69 7.27 CDCl3 7.22 7.20 4.20 4.18 4.17 3.49 3.41 3.40 3.26 3.25 3.24 3.21 3.20 3.19 2.27 2.25 1.86 1.84 1.84 1.81 1.79 1.78 1.76 1.75 1.74 1.67 1.65 1.63 1.62 1.59 1.59 1.56 1.55 1.54 1.54 1.52 1.50 1.49 1.48 1.47 1.46 1.43 1.42 1.40 1.38 1.35 1.33 1.26 1.25 1.16 1.15 1.13 1.12 1.10 1.09 1.08 1.06 1.05 1.02 1.01 0.98 0.96 0.95 0.92 0.91 0.89 0.88 0.87 0.86 0.86 0.68 0.00 -0.00
0.88 0.98 0.95 0.93 1.00 0.95 0.91 1.88 2.00 1.10 7.20 1.82 3.01 2.29 7.01 16.07 1.68 4.45 4.03 14.99 2.86
f1 (ppm)

CND19

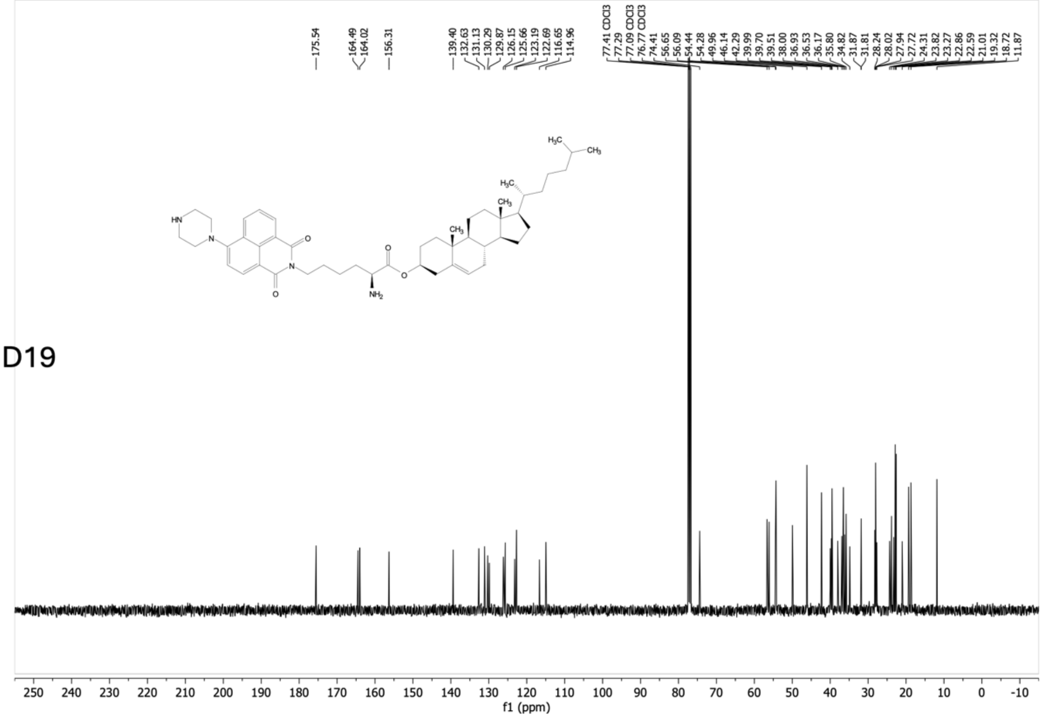
175.54 164.49 164.02 156.31 139.40 132.63 131.13 130.29 129.87 126.15 125.66 123.19 122.69 116.65 114.96 77.41 CDCl3 77.29 77.09 CDCl3 76.77 CDCl3 74.41 56.65 56.09 54.44 54.28 49.96 46.14 42.29 39.99 39.70 39.51 38.00 36.93 36.53 36.17 35.80 34.82 31.87 31.81 28.24 28.02 27.94 27.72 24.31 23.82 23.27 22.86 22.59 21.01 19.32 18.72 11.87
f1 (ppm)

CND19

KI

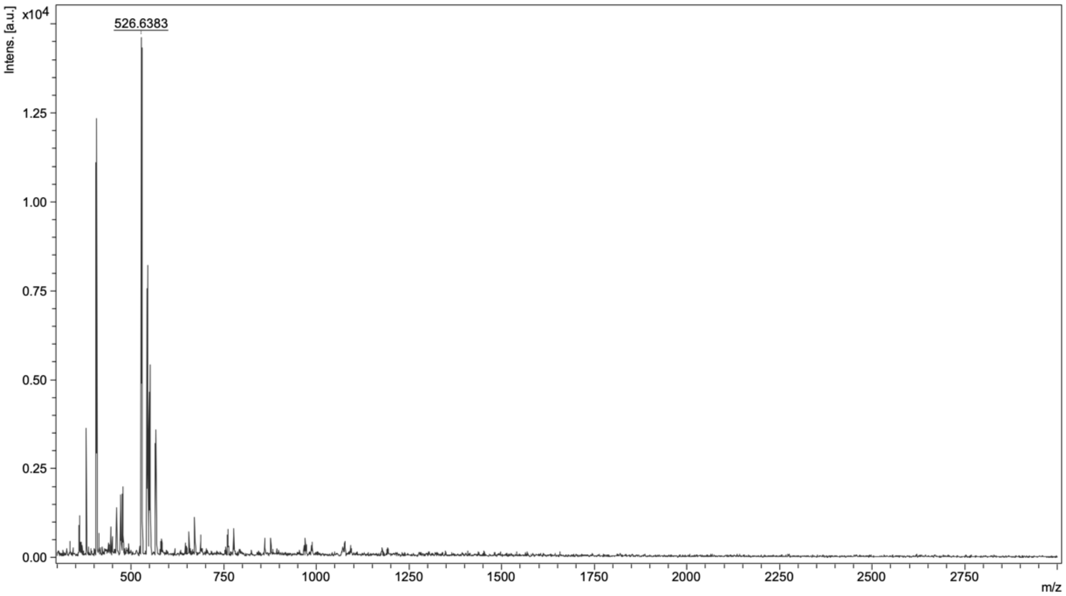

Intens. [a.u.]
x10^4
526.6383
1.25
1.00
0.75
0.50
0.25
0.00
500
750
1000
1250
1500
1750
2000
2250
2500
2750
m/z


KII

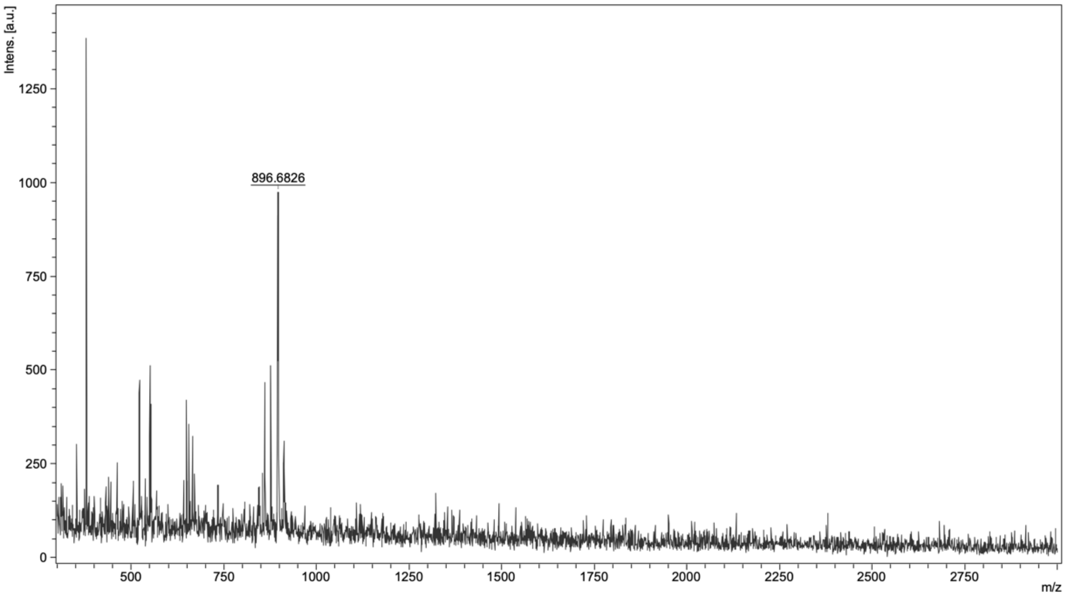

Intens. [a.u.]
896.6826
1250
1000
750
500
250
0
500
750
1000
1250
1500
1750
2000
2250
2500
2750
m/z

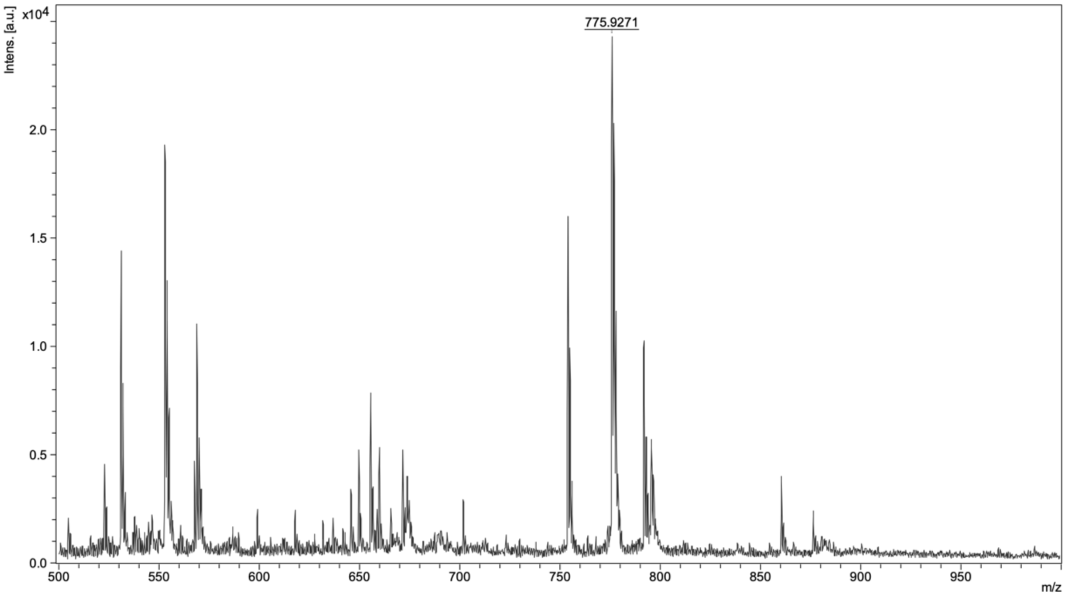
Intens. [a.u.]
x10^4
775.9271
2.0
1.5
1.0
0.5
0.0
500
550
600
650
700
750
800
850
900
950
m/z

CND15

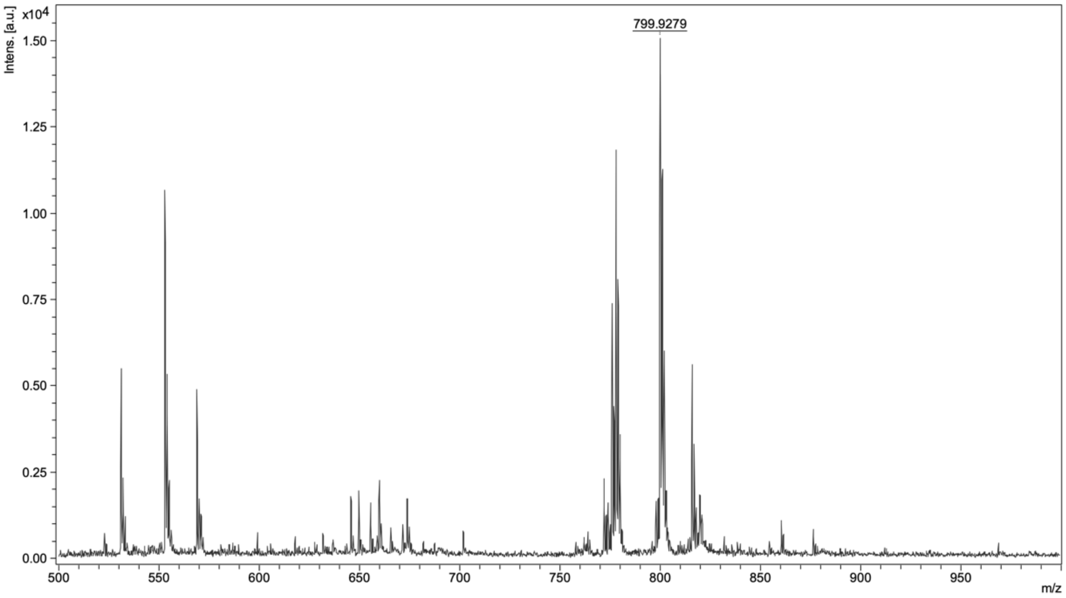
Intens. [a.u.]
x10^4
799.9279
1.50
1.25
1.00
0.75
0.50
0.25
0.00
500
550
600
650
700
750
800
850
900
950
m/z

CND16

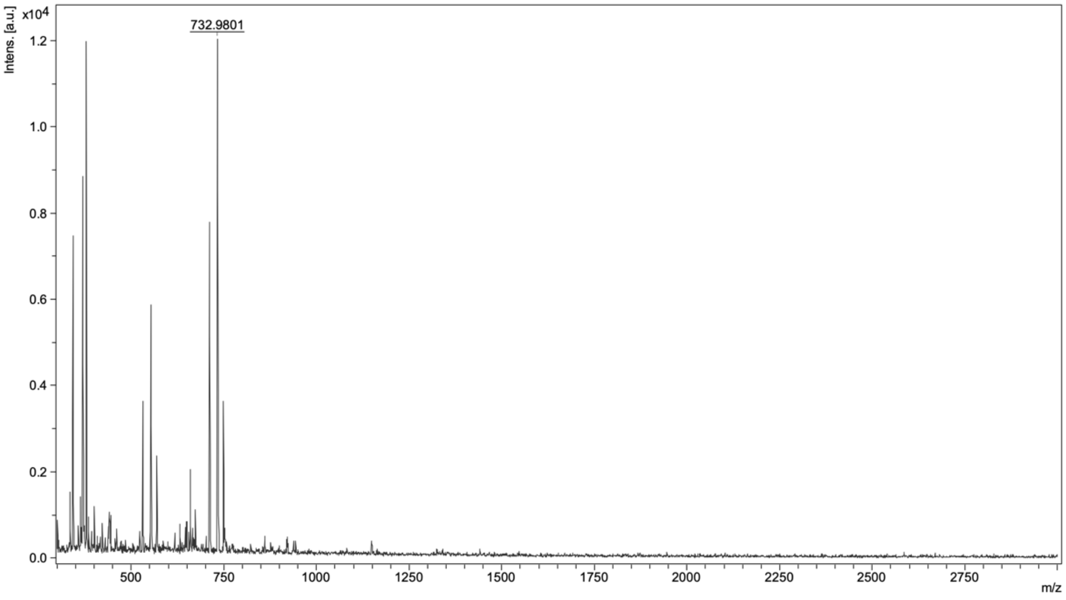
x10⁴
Intens. [a.u.]
732.9801
1.2
1.0
0.8
0.6
0.4
0.2
0.0
500
750
1000
1250
1500
1750
2000
2250
2500
2750
m/z

CND17

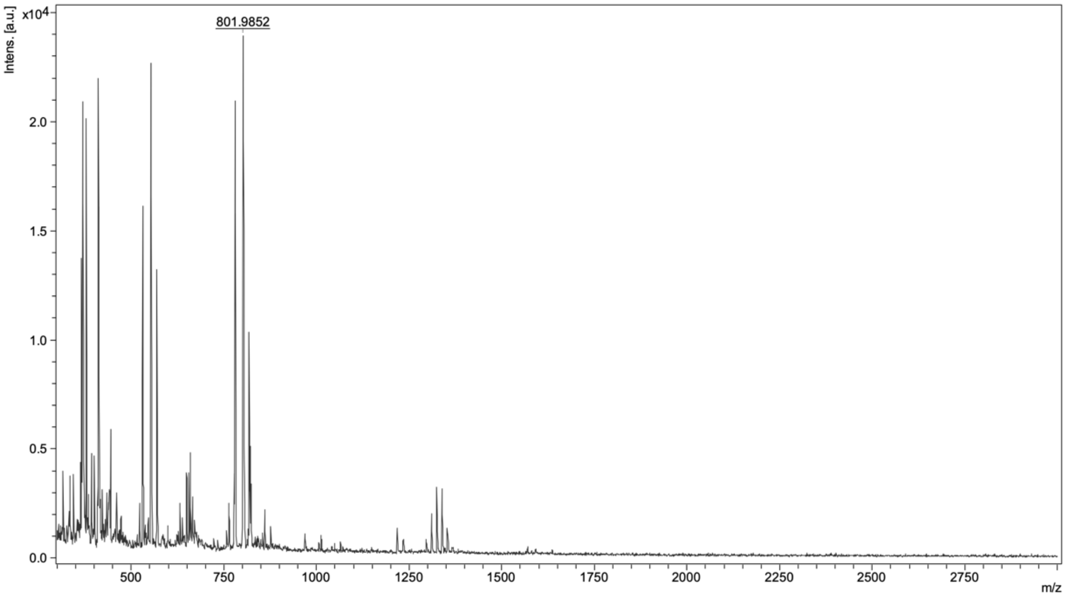
x10⁴
Intens. [a.u.]
801.9852
2.0
1.5
1.0
0.5
0.0
500
750
1000
1250
1500
1750
2000
2250
2500
2750
m/z

CND18

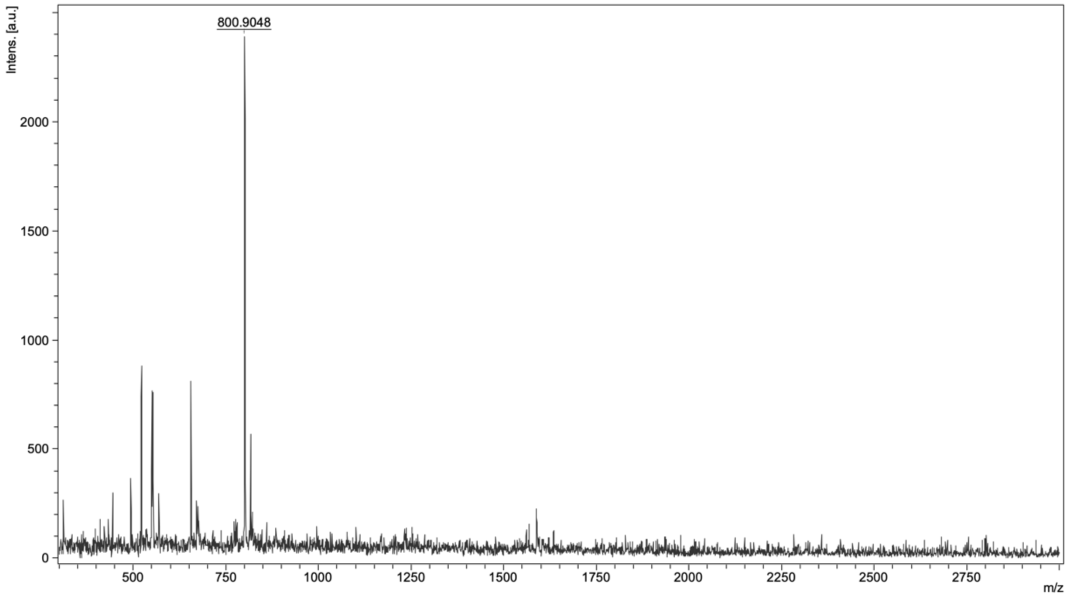
800.9048
Intens. [a.u.]
2000
1500
1000
500
0
500
750
1000
1250
1500
1750
2000
2250
2500
2750
m/z
CND19
